# Spatially segregated APOE^+^ macrophages restrict immunotherapy efficacy in clear cell renal cell carcinoma

**DOI:** 10.7150/thno.109097

**Published:** 2025-04-13

**Authors:** Qintao Ge, Jialin Meng, Zhongyuan Wang, Aihetaimujiang Anwaier, Jiahe Lu, Xi Tian, Yue Wang, Jianfeng Yang, Hailiang Zhang, Dingwei Ye, Wenhao Xu

**Affiliations:** 1 Department of Urology, Fudan University Shanghai Cancer Center; Center; Department of Oncology, Shanghai Medical College; Qingdao Institute of Life Sciences, Fudan University, Shanghai, 200032, P.R. China.; 2 Shanghai Genitourinary Cancer Institute, Shanghai, 200032, P.R. China.; 3 Department of Urology, The First Affiliated Hospital of Anhui Medical University, Hefei, 230022, P.R. China.; 4 Institute of Urology, Anhui Medical University, Hefei, 230022, P.R. China.; 5 Anhui Province Key Laboratory of Genitourinary Diseases, Anhui Medical University, Hefei, 230022, P.R. China.; 6 Department of Urology, Longhua Hospital, Shanghai University of Traditional Chinese Medicine, Shanghai, 200032, P.R. China.; 7 Department of Urology, Fudan University Shanghai Cancer Center, P.R. China.; 8 Department of Urology, Longhua Hospital, Shanghai University of Traditional Chinese Medicine, P.R. China.

**Keywords:** APOE+ macrophages, Immune checkpoint blockade (ICB), Tumor microenvironment (TME), Clear cell renal cell carcinoma (ccRCC), Immune resistance

## Abstract

**Background:** Immunotherapy has revolutionized cancer treatment and holds great potential for them, including metastatic clear cell renal cell carcinoma (ccRCC). However, immune resistance remains a major obstacle, limiting its efficacy and durability. Understanding the mechanisms of immune tolerance in the tumor microenvironment (TME) is pivotal for overcoming these challenges and enhancing therapeutic outcomes.

**Methods:** Over 2000 samples, including a real-world cohort of 230 advanced ccRCC patients treated with immune checkpoint blockade (ICB) were analyzed. Single-cell RNA sequencing data from 13 tumor regions were categorized into ICB-exposed, ICB-resistant, and ICB-responsive groups. Multiple robust algorithms and multiplex immunofluorescence were used to explore TME composition and macrophage heterogeneity. Spatial communication dynamics were further investigated. *In vitro* experiments were performed to evaluate the impact of SPP1 on 786-O and 769-P cells. Co-culture experiments with THP-1-derived macrophages, followed by Western blot, flow cytometry, and functional assays, were performed to investigate SPP1-mediated macrophage polarization and its impact on tumor progression.

**Results:** The results revealed an elevated presence of Apolipoprotein E (APOE)^+^ macrophages in ICB-resistant ccRCC. Notably, higher APOE^+^ macrophage proportion indicated shorter prognosis and worse response to ICB (*P* < 0.001). Elevated expression of CCAAT Enhancer Binding Protein Delta (CEBPD) was markedly linked to several immunosuppressive pathways, hindering T cell recruitment, promoting exhaustion, ultimately diminishing poorer prognosis and worse ICB efficacy. Meanwhile, upregulated Secreted Phosphoprotein 1 (SPP1) significantly enhances the proliferation, clonal formation, and migration of ccRCC cells. Tumor-derived SPP1. Additionally, SPP1 signaling from malignant cells appeared to recruit APOE^+^ macrophages to tumor margins, and promotes macrophage polarization into APOE^+^ M2-like macrophages. In the vicinity of the tumor, these APOE^+^ macrophages shape immunosuppressive TME by releasing abundant TGF-β signals, limiting anti-tumor effector T cells activity in ICB-resistant tumors, and contributing to tumor progression.

**Conclusion:** This study reveals the critical role of APOE^+^ macrophages in promoting immune suppression and resistance to ICB therapy in ccRCC. By promoting T cell exhaustion and immunosuppressive signaling, particularly via localized TGF-β, these spatially segregated macrophages undermine treatment efficacy. Targeting APOE^+^ macrophages, especially in conjunction with ICB, presents a promising strategy to overcome immune resistance and enhance outcomes for ccRCC patients.

## Introduction

Renal cell carcinoma (RCC) is listed among the top ten most common malignant cancers worldwide, representing 4.2% of all new cancer diagnoses. The most common histological subtype, clear cell renal cell carcinoma (ccRCC), originates from the proximal renal epithelial tubules and leads to the majority of deaths related to cancer 1-3. Significant challenges exist in the early detection of this disease, with around 30% of ccRCC patients being diagnosed at presentation with distant metastasis. In recent years, the adoption of targeted therapies and immunotherapy in clinical practice has resulted in a slight increase in the overall survival rate for patients 4. Nevertheless, variances in individual treatment responses and the development of drug resistance mean that most individuals eventually progress to the advanced ccRCC stage, which has a 5-year survival rate of under 10% 5, 6.

Immunotherapy that utilizes immune checkpoint inhibitors has surfaced as a promising strategy for treating various cancers by rejuvenating T cell activity and promoting the destruction of tumor cells 7. Currently, a significant challenge limiting the widespread application of immune checkpoint blockade treatment (ICB) in ccRCC is the lack of robust and reliable biomarkers to predict therapeutic efficacy.

According to the National Comprehensive Cancer Network (NCCN) guidelines, ICB is recommended as the first-line adjuvant treatment for high-risk renal cancer that defined by the International Metastatic RCC Database Consortium (IMDC) risk score 8, while only a small percentage of patients have realized substantial and lasting benefits 9, 10. A subset of ccRCC patients experience rapid disease progression following ICB treatment, with approximately 20-30% demonstrating primary resistance or quick relapse post-therapy, underscoring the critical issue of immune tolerance. Consequently, understanding the mechanisms driving such resistance is crucial to improving patient outcomes and expanding the utility of ICB 11.

The tumor microenvironment (TME) is highly heterogeneous, providing a complex ecosystem that can profoundly influence both tumor progression and therapeutic resistance. This heterogeneity offers a valuable window for dissecting the intricate interplay between tumor cells and immune components, particularly in understanding how these interactions evolve over time and under therapeutic pressure 12, 13. Investigating the phenotypes and functions of specific immune cell populations infiltrating tumors at various stages of ccRCC progression or in response to ICB is vital. Such studies can uncover key molecular events that drive immune cell exhaustion, immune evasion, and impaired anti-tumor responses 14-16. Moreover, identifying immune-regulatory signals and biomarkers associated with tumor evolution holds significant potential for developing novel drug targets and sensitization strategies for anti-tumor immunotherapy 17, 18. These insights could also pave the way for new technologies to monitor tumor progression, therapeutic response, and prognosis through peripheral immune detection, ultimately advancing our understanding of immune modulation and tumor evolution of ccRCC.

The role of tumor-associated macrophages (TAMs) in regulating tumor immunity and influencing responses to ICB therapy has been reported in previous studies 19. Macrophages are traditionally characterized by a dual role in cancer immunotherapy, with M1-like macrophages displaying anti-tumor activity, while M2-like macrophages promote tumor growth, metastasis, and immune evasion. In the TME, TAMs predominantly exhibit an M2-like phenotype, contributing to tumor progression and resistance to ICB therapy 20, 21. M2-like TAMs have been widely recognized for their critical role in the immunosuppressive environment of tumors. These TAMs can directly inhibit T cell activity by expressing high levels of surface molecules, including PD-L1 22, CD206 23, and CD163, which interact with T cell molecules such as PD-1, leading to immune evasion. Additionally, TAMs secrete chemokines, such as CCL2, CCL5, and IL-10, which recruit and induce other immunosuppressive cells, including regulatory T cells (Tregs) 24, 25, thereby further suppressing anti-tumor immune responses. Additionally, our further work, patients belong to early-TLS (tertiary lymphoid structures) group harbored more CD68 ^+^ CD163 ^+^ M2 infiltration. The presence of early-TLS in the former is thought to be associated with poor-prognosis, suggesting the significant role of TAMs in cancer progression 26.

Recent advancements in single-cell and spatial transcriptome sequencing have enabled deeper characterization of macrophage subtypes with distinct functional attributes. For instance, Bill *et al.* identified macrophages characterized by CXCL9 and SPP1 expression to better elucidate the polarization and prognostic implications 27. Ma* et al.* explored the transcriptomic diversity of TAMs at a single-cell level, categorizing them into seven distinct subtypes by unique functional markers, including lipid metabolism (*APOE, APOC1, ACP5, FABP5*), angiogenic factors (*VEGFA, SPP1*), IFN regulation (*IDO, ISG15, CXCL8, CXCL9, CXCL10*), and cell cycle progression (*MKI67, CDK1*) 28. These TAM subsets play critical roles in driving tumor progression, shaping the TME, and mediating varied responses to ICB therapy.

A pan-cancer analysis has emphasized the pivotal role of APOE^+^ macrophages in influencing anti-tumor immunotherapeutic outcomes 29. Notably, non-responders to ICB therapy exhibit higher infiltration of APOE^+^ macrophages compared to responders. Additionally, responders tend to have a greater spatial separation between APOE^+^ macrophages and CD8^+^ T effector exhausted cells. Preclinical studies have also demonstrated that combining APOE inhibitors with ICB therapy significantly enhances therapeutic efficacy in mouse models. These findings suggest that the presence of APOE^+^ macrophages is associated with poor response to ICB therapy, highlighting their potential as therapeutic targets to improve treatment outcomes.

The importance of APOE^+^ macrophages in ccRCC remains poorly understood. Specifically, the effects of APOE^+^ macrophages on ccRCC tumor progression, their contribution to reshaping the immune microenvironment, and their impact on immunotherapy and underlying mechanisms are not yet fully elucidated. To investigate these, we first employed single-cell spatial multi-omics and multiplex immunofluorescence (mIF) to characterize the infiltration patterns of APOE^+^ macrophages within tissues and constructed a target gene network integrated with spatial cell-cell communication analysis, revealing their potential role in shaping the TME. Based on these findings, we conducted functional analyses, including *in vitro* transfection, colony formation assays, CCK-8 proliferation assays, and Transwell migration/invasion assays, to confirm the role of SPP1 in the progression of ccRCC. Furthermore, SPP1-based co-culture experiments combined with Western blot, ELISA, and flow cytometry demonstrated that tumor-derived SPP1 regulates macrophage phenotypes and contributes to the immunosuppressive function of APOE^+^ macrophages.

## Method and Materials

### Data collection and processing

Single-cell RNA sequencing (scRNA-seq) data (PRJNA705464) 30 were downloaded from the European Nucleotide Archive (ENA) database (https://www.ebi.ac.uk/ena/browser/home). This cohort comprised six patients with advanced disease, from whom different tumor regions and peripheral blood mononuclear cells were subjected to single-cell sequencing, encompassing proximal, distal, and central tumor areas (N = 29). The study primarily focuses on the tumor itself and its surrounding microenvironment; therefore, only 13 samples from three patients with varying immunotherapy responses were selected. Data from different tumor regions of each patient were analyzed, categorizing them into the Nivo-exposed group, Ipi/Nivo-resistant group, and Ipi/Nivo-completely remitted group, which correspond to the ICB-exposed group, ICB-resistant group, and ICB-responsive group, respectively. The subsequent analysis focused on single-cell sequencing data from 13 tumor regions (N = 13). Additionally, spatial transcriptome sequencing data (GSE210041) were obtained from the GEO database (https://www.ncbi.nlm.nih.gov/geo/) 31. Bulk sequencing data and clinical profiles were sourced from TCGA-KIRC (N = 530) and EMTAB3267 (N = 53), accessible via The Cancer Genome Atlas (TCGA, https://portal.gdc.cancer.gov/) and ArrayExpress (https://www.ebi.ac.uk/arrayexpress/). The Tumor Immune Dysfunction and Exclusion (TIDE, http://tide.dfci.harvard.edu/) algorithm was used to predict potential responders to immunotherapy in the two cohorts 32. Three external immunotherapy cohort included GSE67501 (N = 11) 33, and Checkmate cohorts (N = 311) 16, Miao* et al.* (N = 16) 34, which were acquired from the GEO database and previously published articles. A real-world ICB therapy cohort, FU-ICI (N = 230), were also used, and more detail could refer to prior studies 35. The gene sequencing outcomes across the three groups were represented as transcripts per million (TPM), where mRNAs with a TPM value of less than 1 were present in more than 90% of the samples that were not included in the analysis. Additionally, patients without corresponding mRNA profiles, clinical data, or follow-up timelines were eliminated to reduce the likelihood of bias. Overall survival (OS) and progression free interval (PFS) were set as clinical outcome parameters.

### Quality control, clustering and annotation of single-cell data

For the processed 10x data, the R package Seurat version 4.3.0 was employed for the initial preprocessing stages 36. Double cells were removed using the R package DoubletFinder 37 and inferior cells were filtered. Exclusion criteria: 1) per cell with detected a gene number > 6000 or < 200; 2) The proportion of mitochondrial gene count more than 10%. In the gene filtration step, any genes expressed in fewer than five cells were disregarded. The function CellCycleScoring() was employed to assess the cell cycle status, and subsequently, the regressout algorithm within the ScaleData() function was applied to reduce the influence of cell cycle variations. Following the normalization and scaling of expression data, batch effects were addressed using the R package Harmony, which also facilitated the selection on of the top 2000 variable genes. Principal Component Analysis (PCA) was applied to the top 2000 highly variable genes to achieve dimensionality reduction of the data. By extracting the principal components that account for the greatest variability, PCA reduces dimensionality while retaining the majority of important biological information. Subsequently, based on the first 15 principal components, the Leiden algorithm was employed to identify cell populations by optimizing modularity. Finally, the UMAP algorithm was utilized to further reduce dimensionality, generating a two-dimensional embedding that optimizes the local structural relationships between data points. The annotation of the clustered cell populations was conducted manually by referencing signature genes associated with various cell types and consulting relevant literature 38, 39. Visualization of the data was executed using the SCP R package (available at https://github.com/zhanghao-njmu/SCP) alongside the omicverse Python module 40.

### BayesPrism deconvolution analysis

In this research, we employed the BayesPrism algorithm to separate bulk RNA sequencing data of EMTAB3267 and TCGA-KIRC cohorts into its individual cell types. BayesPrism is a probabilistic, model-driven framework tailored for accurately deconvoluting bulk gene expression data by utilizing reference single-cell RNA sequencing datasets. This approach integrates a Bayesian model that effectively manages the noise found in bulk expression data as well as the uncertainty that is intrinsic to single-cell reference datasets 41.

### TFvelco and PAGA analyses

The TFvelo algorithm was used to examine the dynamic behavior of transcription factors (TFs) by leveraging single-cell RNA velocity data. By combining RNA velocity with recognized TFs regulatory networks, TFvelo allows for the inference of temporal variations in TFs activity throughout cellular processes. We employed TFvelo on our single-cell RNA-seq dataset, using default settings to model the temporal dynamics of TFs. Prior to this, the RNA velocity data underwent preprocessing, and regulatory interactions were established based on known relationships between TFs and their targets. This approach provided us with the capability to monitor shifts in TFs activity across different cellular states, offering valuable insights into the mechanisms of transcriptional regulation. The analysis was carried out using the TFvelo package in the Python.^42^. In addition, Partition-based graph abstraction (PAGA) analysis was performed by RunPAGA() implemented in SCP package to infer connectivity and potential lineage relationships between cell clusters in single-cell RNA sequencing data 43.

### Infercnv analysis

Epithelial cells were isolated, and a novel gene-cell matrix was created. The somatic large-scale chromosomal copy number variation (CNV) score for each ductal cell was computed with the use of the R package inferCNV (v1.6.0). Following the data requirements specified in the inferCNV documentation (https://github.com/broadinstitute/inferCNV), we assembled a raw counts matrix, an annotation file, and a gene/chromosome position file. Normal epithelial cells were used as reference normal cells. The analysis with inferCNV began with parameters, including 'denoise,' default hidden Markov model (HMM) settings, and a cutoff threshold of 0.1. To reduce the likelihood of erroneous CNV identifications, the default Bayesian latent mixture model was applied to ascertain the posterior probabilities of CNV changes in each cell, adopting a standard threshold value of 0.5 44.

### Construction of Regulon network and AUCell analysis

The SCENIC package (version 1.2.4) 45 was utilized to build the gene regulatory network. This undertaking encompassed an examination of 38 transcription factor (Regulon) motif enrichment and co-expression modules derived from the dataset. Furthermore, the AUCell package (version 3.12) was used to calculate and rank the activities of Regulons based on their Regulons specificity scores. GENIE3 facilitated the identification of co-expressed genes related to each Regulon, and Spearman's correlation was then applied to determine the relationships between Regulons and their target genes. Using the findings from the co-expressed genes associated with each TF, we developed the Regulon-target gene network, which was subsequently annotated with references to GO and KEGG pathways. To compare the functional status of T cells across different groups, we conducted an AUCell score analysis for various genes associated with cytotoxicity (including *GZMB, GZMH, GZMK, GZMA, TIA1, PRF1, LAMP1, GNLY, FASLG, SLAMF7, ZAP70, CD69, TNF*) and exhaustion (comprising *LAG3, PDCD1, TIGIT, HAVCR2, CD160, CTLA4*).

### Cell-to-cell communication analysis in single-cell analysis

To understand the communication between cells and the interaction from ligand to receptor (L-R), we conducted an analysis using Cellchat (v.2.0) 46. We analyzed the distinct communication characteristics between the ICB-resistant group and the ICB-exposed group using the CellChatDB.human database. Intercellular communication for each signaling pathway was established through the computeCommunProbPathway() function, while graphical representations were generated using the netVisual_chord_gene() function. The inference of intercellular communication networks was conducted with default parameters. The rankNet function was employed to evaluate the overall information flow, which allowed for the identification of signals exhibiting significant differences in activity between the two groups. For each specific pathway, we calculated the network centrality measures for each cell group, identifying the dominant senders, receivers, mediators, and influencers within the cell-cell communication network. For more details, please refer to the tutorial documentation (https://github.com/sqjin/CellChat/tree/master/tutorial).

### Spatial transcriptome analysis

The R package Seurat was employed to carry out QC, clustering, and gene expression analysis on the acquired spatial transcriptomics (ST) data. All analyses were conducted on a representative ST sample (GSM6415705, Stage IV, ISUP3) from the GSE210041 dataset. Mitochondrial and ribosomal genes were excluded to minimize technical noise. Low-abundance genes, defined as those expressed in fewer than 10 spots, were filtered out to ensure robust expression data. Data normalization was performed using SCTransform to stabilize variance across spots. PCA was conducted on the top 3,000 variable genes, and the first 20 principal components were selected for downstream analysis. To analyze the spatial arrangement of the cell subclusters detected from the single-cell cohort, the ST and single-cell RNA-sequencing expression matrices were merged and co-dimensioned through the use of Robust Cell Type Decomposition (RCTD) 47. RCTD utilizes a probabilistic approach that incorporates scRNA-seq data as a reference to estimate the composition of various cell types within each spatial transcriptomics spot. This technique represents gene expression as a weighted combination of cell types, considering both variation specific to genes and technical noise present in the spatial data. The investigation of cell-to-cell interactions and ligand-receptor (L-R) identification was performed with Stlearn algorithms 48. The spatial map illustrating cell dependencies was generated using the MISTy algorithms implemented in the MISTy (v1.2.1) package 49. The estimations of cell types derived from RCTD across all slides were amalgamated into a cohesive model utilizing two different spatial contexts: (1) an intrinsic context, evaluating correlations among deconvolution estimates in a particular location; (2) a juxta context, which consolidates deconvolution estimates from adjacent spots within a maximum distance of 5. The aggregated standardized importance values (median) from each context across all slides were interpreted as spatial relationships between cell types, such as co-localization or mutual exclusion, although this does not suggest any causal connections. Before aggregation, predictors displaying an R² value below 10% for the target cell type were omitted from each slide.

### Immunohistochemical (IHC) staining analysis

Tissue chip from 230 individuals 35 who had undergone either radical or partial nephrectomy at the Department of Urology, Fudan University Shanghai Cancer Center (FUSCC, Shanghai, China), were chosen for this analysis. We conducted IHC staining to evaluate the expression of APOE (anti-APOE antibody: Cat. PA5-27088, ThermoFisher, USA) and PD-L1 (anti-PD-L1 antibody: Cat. DF6526, RRID: AB_2838488, Affinity, USA) and CEBPD (anti-CEBPD antibody: Cat. AF9027, RRID: AB_2843218, Affinity, USA) between ICB-response and ICB-resistant tissues. Detailed IHC procedures could refer to our prior studies 50, 51. Tumor samples were gathered and preserved in a 4% formaldehyde solution for 24 h. Subsequently, these samples were embedded in paraffin and sectioned into approximately 5 μm thick slices. The tumor sections underwent deparaffinization and rehydration, followed by the inhibition of endogenous peroxidase activity and antigen retrieval. After that, a 5% BSA solution was applied to the tumor sections to minimize non-specific binding for 30 min, after which they were incubated with primary antibodies overnight. Following a secondary antibody incubation for one hour, the tumor sections were visualized using a DAB kit.

### Multiplex immunofluorescence analysis

Multiplex immunofluorescence (mIF) was performed to evaluate the spatial distribution and co-expression of specific markers within the tumor microenvironment. Formalin-fixed, paraffin-embedded (FFPE) tissue sections were subjected to sequential staining using the Opal™ multiplex immunofluorescence system (Akoya Biosciences). The following primary antibodies were employed: CD68 (macrophage marker), CD163 (M2 macrophage marker), APOE (apolipoprotein E), and CK (cytokeratin, epithelial marker). DAPI (4',6-diamidino-2-phenylindole) was used for nuclear counterstaining. After deparaffinization and rehydration, antigen retrieval was performed using Tris-EDTA buffer (pH 9.0) in a pressure cooker. The tissue sections were blocked with a protein blocking buffer to reduce non-specific binding. Each primary antibody was applied sequentially, followed by the corresponding Opal fluorophore-conjugated secondary antibody. Between each staining round, antigen retrieval was repeated to strip off the previous antibody complex without affecting the fluorophore conjugates 26, 35. The fluorophores used were as follows: Opal 520 for CD68, Opal 570 for CD163, Opal 620 for APOE, and Opal 690 for CK. Finally, DAPI was added to visualize the nuclei.

The stained slides were imaged based on the Vectra Polaris™ imaging system (Akoya Biosciences) under multispectral conditions. Images were captured at 20× magnification, and the fluorescence signals for each marker were unmixed into individual channels using inForm software (Akoya Biosciences). This method allowed for the precise localization and quantification of the various immune cell populations and their interactions with tumor cells, providing critical insights into the tumor microenvironment.

### Cell lines culture and transfection

Human ccRCC lines 786-O and 769-P were cultured in RPMI-1640 medium supplemented with 10% fetal bovine serum (FBS) and 1% penicillin/streptomycin. To knock down the expression of SPP1, two independent shRNA vectors (SPP1 Sh1 and Sh2) and a control shRNA (shControl) were transfected using Lipofectamine 3000. For the overexpression of SPP1 (SPP1 OE), the SPP1 expression vector or an empty vector control was transfected. SPP1 Sh-1: CCGAGGTGATAGTGTGGTTTA, SPP1 Sh-2: CCACAAGCAGTCCAGATTATA.

### Colony formation analysis

The assay for colony formation involves the initial seeding of cells in a 6-well plate, followed by a culture period lasting 14 days. After this incubation phase, 4% polyformaldehyde is utilized to fix the colonies, which are then stained with crystal violet and counted manually. The quantities of colonies from various experimental groups are measured and analyzed to derive pertinent conclusions.

### CCK-8 assay analysis

The assessment of cell proliferation is conducted using the CCK-8 assay technique. Cells are placed in a 96-well plate and treated with the CCK-8 solution at specific time intervals of 24, 48, 72, and 96 h. To determine cell proliferation, the absorbance is measured at a wavelength of 450 nm.

### Transwell invasion assay

For conducting the transwell invasion assay, a 24-well Transwell chamber equipped with an 8.0 μm pore polycarbonate membrane insert is employed. Initially, 50 μL of a diluted Matrigel solution (mixed at a ratio of 1:8 in serum-free medium) is used to coat the upper chamber, which is then incubated at 37°C for 2 hours. The 786-O and 769-P cell lines are treated with trypsin, reconstituted in serum-free medium, and adjusted to a concentration of 1 × 10⁵ cells per 200 μL prior to being placed in the upper chamber. To act as a chemotactic agent, the lower chamber is filled with 600 μL of complete medium containing 10% FBS. Following this, the cells are incubated at 37 °C with 5% CO₂ for 24 h. Once incubation is complete, a cotton swab is employed to eliminate non-invading cells from the upper chamber, and the membrane is subsequently washed with PBS. The cells that have invaded and are attached to the lower surface are fixed using 4% paraformaldehyde for 15 min, stained with 0.1% crystal violet for an additional 15 min, washed again with PBS, and left to air dry. The number of invading cells is then counted in 5 randomly chosen fields within each well, using an optical microscope set at 100× magnification.

### Wound healing assay

Inoculate 786-O and 769-P cells into 6-well plates and culture them in complete medium until they reach 90-100% confluence. Use a 10μL pipette tip to create a straight wound line on the cell monolayer. Gently wash the wells with PBS to remove any detached cells, and then add fresh serum-free medium to minimize the impact of cell proliferation. Capture images using an inverted microscope at 0 h and 24 h. Utilize ImageJ software to measure the wound area at each time point to quantify the wound closure rate. All experiments are conducted in triplicate.

### Co-culture analysis

THP-1 monocytes were differentiated into M0 macrophages by priming with 100 nM PMA for 48 h, followed by co-culture with three experimental groups: (i) Ordinary group (macrophages in standard RPMI-1640 medium with 10% FBS), (ii) Vetor co-cultured condition medium (CM) group (macrophages co-cultured with vector-transfected cells), and (iii) SPP1 OE co-cultured CM group (macrophages co-cultured with SPP1 OE renal cancer cells). Furthermore, in further phenotypic analysis, three groups were divided, including: (i) Vetor co-cultured CM group (macrophages co-cultured with vector-transfected cells), (ii) SPP1 OE co-cultured CM group (macrophages co-cultured with SPP1 OE renal cancer cells), and (iii) SPP1 OE co-cultured CM + APOE Ab group (macrophages co-cultured with SPP1 OE renal cancer cells and APOE antibody). All CM were centrifuged (2000 rpm, 10 min) to remove debris, sterilized through 0.22 μm membranes, and subjected to cross-lineage validation (e.g., 786-O-derived CM applied to 769-P) to eliminate cell type-specific bias.

### Western Blot (WB) analysis

Cells were lysed with RIPA buffer containing protease inhibitors. The protein concentration was determined using a BCA kit. Proteins were separated by SDS-PAGE and transferred onto PVDF membranes. After blocking with 5% non-fat milk, the membranes were incubated with primary antibodies overnight at 4 °C, followed by secondary antibodies for 1 h at room temperature. Protein bands were visualized using ECL reagents and analyzed with ImageJ software.

### Flow cytometry analysis

Macrophages were incubated for 48 h in either vector Co-cultured CM or SPP1 OE co-culture CM. After the treatment, the cells are harvested by gentle digestion with trypsin, followed by two washes with ice-cold PBS, and then resuspended in FACS buffer, which consists of PBS with 2% FBS. For the staining of surface markers, the cells need to be incubated with fluorochrome-conjugated antibodies targeting CD206-PE, APOE-APC, and PDL1-FITC at 4 °C in the dark for 30 min. Following this incubation period, the cells should be washed twice with FACS buffer and then resuspended in 500 μL of PBS before proceeding with the analysis. Flow cytometry was performed using a BD FACSCanto II flow cytometer, with the data analyzed using FlowJo software. The quantification of positive cells for each marker was accomplished following the established gating strategy.

### ELISA analysis

The cell culture supernatant was collected, centrifuged at 1000 × g for 10 min at 4 °C, and stored at -80 °C. Cytokine levels (CCL2, TGF-β, and IL-10) were measured using an ELISA kit according to the manufacturer's instructions. Briefly, a 96-well plate was pre-coated with a capture antibody, blocked with 1% BSA, and then incubated with either the supernatant or standard for 2 h. After washing, biotin-labeled detection antibody and HRP-streptavidin were added, followed by the addition of TMB substrate for color development. The absorbance was measured at 450 nm. All experiments were performed in triplicate.

### Statistical analysis

The analysis of categorical data was performed using Fisher's exact test and the rank sum test. For comparisons between two groups, a T-test was employed, whereas ANOVA facilitated pairwise comparisons among several groups. K-M curves were plotted for survival analysis. All statistical analyses were performed using, R (Version: 4.2.2) and Python (Version: 3.9). A two-tailed *p*-value < 0.05 was recognized as statistically significant.

## Results

### APOE^+^ macrophage enrichment correlates with immunotherapy resistance in ccRCC

**Figure 1:**
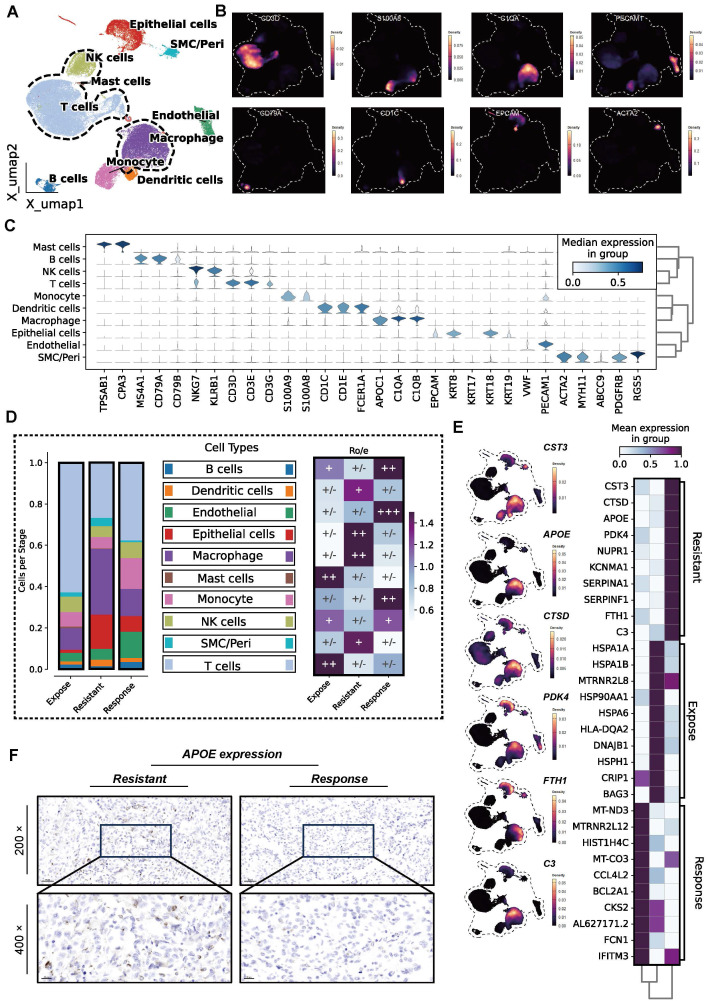
** Increased APOE^+^ macrophages in immune checkpoint blockade (ICB)-resistant tumors.** (**A**) Uniform Manifold Approximation and Projection (UMAP) visualization of ten distinct cell populations. (**B**) Density plot showing the expression levels of CD3D, S100A8, C1QA, PECAM, CD79A, CD1C, EPCAM, and ACTA2 across 62,840 cells. (**C**) Stacked violin plot illustrating the expression of signature genes across the ten cell types. (**D**) Distribution of ten cell types classified by ICB response status (left) and tissue preference across ICB groups (right). (**E**) Heatmap showing the top 10 differentially expressed genes (DEGs) across ICB groups, along with density plots highlighting CST3, APOE, CTSD, PDK4, FTH1, and C3 expression. (**F**) Immunohistochemistry (IHC) comparison of APOE expression between ICB-resistant and ICB-responsive samples.

In this study, we integrated multi-omics data, including scRNA-seq, ST, bulk RNA-seq, and *in vitro* experiments to comprehensively explore the key immunosuppressive mechanisms in the ICB response of ccRCC (**Figure S1, Table S1**). Firstly, a total of 62, 840 cells from 13 samples were ultimately included to delineate the cellular landscape acorss the ICB-response, ICB-expose, and ICB-resistant groups (**Figure S2A**). Uniform Manifold Approximation and Projection (UMAP) was applied for dimensionality reduction and cell clustering (**Figure S2B**). Based on signature gene expression, ten distinct cell types were identified (**Figure 1A**), including epithelial cells, NK cells, T cells, mast cells, endothelial cells, macrophages, monocytes, dendritic cells, and smooth muscle/pericytes (SMC/peri cells). T cells were characterized by high expression of CD3D, CD3E, and CD3G, while NK cells exhibited elevated expression of NKG7 and KLRB1. B cells predominantly expressed MS4A1, CD79A, and CD79B, and monocytes showed high levels of TPSAB1 and CPA3. Macrophages were marked by increased expression of C1QA and C1QB, and dendritic cells by CD1C and CD1E. Epithelial cells were distinguished by high expression of EPCAM and KRT8, endothelial cells by VWF and PECAM1, and smooth muscle/pericytes by ACTA2, MYH11, ABCC9, PDGFRB, and RG55 (**Figure 1B-C, Figure S2C**).

Among the three groups, the ICB-resistant group exhibited significantly higher proportion of TAMs infiltration, as well as increased tissue preference (**Figure 1D**). TAMs demonstrated a distinct preference for the tumor center, as well as both distal and proximal regions of ICB-resistant tumors, although their proportions varied. This observation suggests a potential link between high TAM infiltration and reduced immunotherapy efficacy (**Figure S2D-E**). To further investigate, we calculated cell correlations across the groups (**Figure S2F**). Differentially expressed genes (DEGs) were identified, and the top 10 were visualized. As shown in **Figure 1E**, genes such as* CST3, CTSD, APOE, PDK4, NURP1, KCNMA1, SERPINA1, FTH1*, and *C3* were among the most highly expressed, particularly in TAMs, in the ICB-resistant group.

Given the special role of APOE^+^ macrophages in influencing immunotherapy outcomes, as previously highlighted in a pan-cancer analysis 29, we conducted IHC to evaluate APOE expression in both ICB-resistant and ICB-responsive patients. The results revealed that ICB-resistant patients exhibited higher levels of APOE expression (85 vs. 145, *P* < 0.05) (**Figure 1F**). To further validate these findings, we analyzed APOE expression in an independent immunotherapy cohort (the Checkmate cohort) (**Figure S2G**), which similarly showed elevated APOE levels in patients who experienced no clinical benefit (NCB) compared to those with clinical benefit (CB) (*P* = 0.02). In FU-ICI cohort (N = 230), we found high expression of APOE correlated to a shorter OS (*P* = 0.002) and PFS (*P* < 0.001) (**Figure S2H**). Patients exhibiting elevated levels of APOE expression were more likely to encounter progressive disease (PD) or stable disease (SD) events compared to those with lower APOE expression levels. This observation suggests that heightened APOE levels may be linked to resistance to immunotherapy (all *P* < 0.05, **Figure S2I**). Based on this evidence, we hypothesize that APOE^+^ macrophages play a central role in the development of ICB resistance in ccRCC.

### APOE^+^ macrophages as key drivers of ICB resistance through lipid metabolic reprogramming

**Figure 2:**
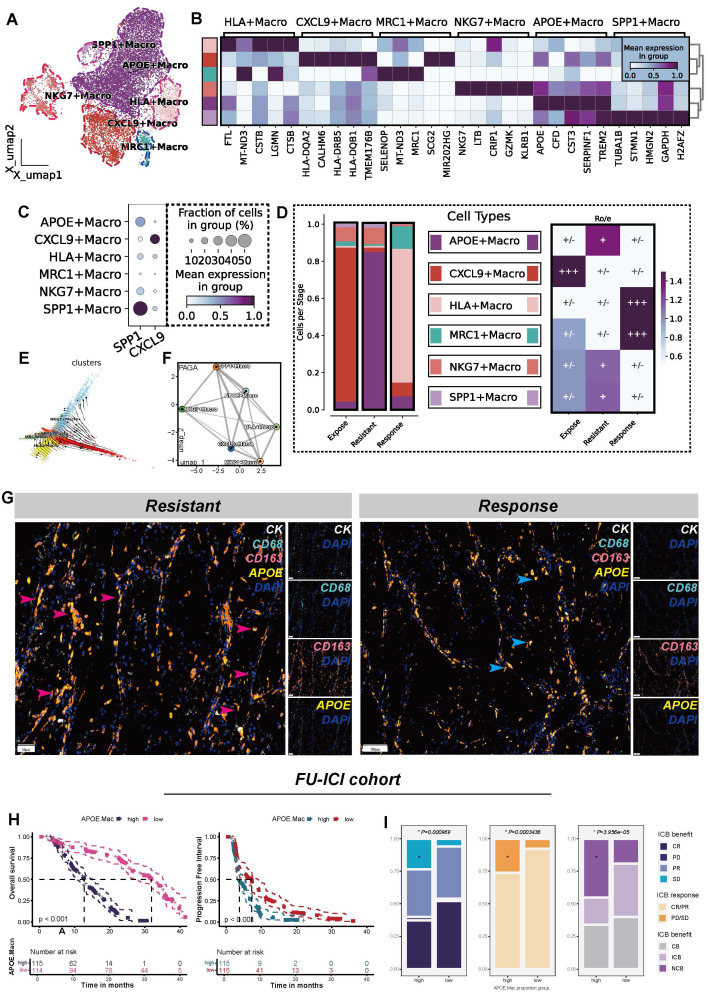
** APOE^+^ macrophages correlate with poor ICB response.** (**A**) UMAP plot illustrating six macrophage populations. (**B**) Top five DEGs across the six macrophages subtypes. (**C**) Comparisons of SPP1 and CXCL9 among the six macrophages. (**D**) Six macrophage distribution classified by ICB groups(left); Tissue preference of six ten populations across ICB groups(right). (**E**) TFvelo analysis. (**F**) PAGA analysis. (**G**) Comparison of cellular components between resistant and response groups assessed by multiplex immunofluorescence (mIF). Red arrows represent high APOE expression in CD68^high^CD163^high^ (M2-like) cells, and blue arrows represent low APOE expression in CD68^high^CD163^low^ (M2-like) cells. CK, cytokeratin (white); DAPI, 4, 6-diamidino-2-phenylindole (mazarine); CD68, Macrophage marker(cycan); CD163, M2-like marker (orange); APOE, Apolipoprotein E(yellow). (**H**) K-M plots demonstrated distinct overall survival (OS) and progression-free survival (PFS) outcomes for groups classified by APOE^+^ macrophage. (**I**) Comparison of ICB response rates between high and low APOE^+^ macrophage proportion groups. CR: complete response; partial response; PR: partial response; PD: progression disease; SD: stable disease.

To validate the hypothesis that APOE^+^ macrophages contribute to ICB resistance, we extracted all macrophages and performed re-clustering. Eventually, this analysis identified six distinct macrophage clusters (**Figure 2A**), and the top 5 differentially DEGs for each macrophages clusters were specifically illustrated in **Figure 2B**. Additionally, in reference to a published study 27, we compared the expression levels of SPP1 and CXCL9 across the six clusters (**Figure 2C**). As a result, six macrophage populations were identified, including APOE^+^ macrophages, CXCL9^+^ macrophages, HLA^+^ macrophages, MRC1^+^ macrophages, and NKG7^+^ macrophages. Notably, SPP1^+^ and APOE^+^ macrophages constituted the majority of macrophages in ICB-resistant tumors. Furthermore, both APOE^+^ and SPP1^+^ macrophages showed a marked preference for the resistant tumor microenvironment (**Figure 2D**).

Further analysis using TFvelo and PAGA algorithms revealed that APOE^+^ macrophages are positioned at the developmental terminal, indicating they represent a terminally polarized macrophage state (**Figure 2E-F**). We found that APOE^+^ macrophages demonstrated high activity in lipid metabolic processes and oxidative phosphorylation pathways (**Figure S3A**), consistent with the characteristics of lipid-associated tumor-associated macrophages (LA-TAMs) 28. Lipid metabolism in macrophages is closely linked to immunosuppression and immune tolerance functions 52, 53, and LA-TAMs are known to actively suppress anti-tumor immune responses while promoting tumor progression 54.

mIF analysis confirmed a significantly greater presence of APOE^+^ macrophages in the ICB-resistant group compared to the immune-responsive group. Notably, many of these macrophages exhibited high expression levels of CD68 and CD163, indicating M2-like macrophages, which are known for their roles in inflammation suppression and tumor progression 55 (**Figure 2G**). To combine phenotypic features of ccRCC patients, we conducted BayesPrim deconvolution algorithm in FU-ICI cohort (N = 230). The results showed that higher APOE^+^ macrophage proportion indicated shorter OS (*P* < 0.001) and PFS (*P* < 0.001) (**Figure 2H**). Meanwhile, despite receiving ICB treatment, patients with high APOE^+^ macrophage infiltration characteristics may be more likely to have disease progression and less likely to benefit from it (all *P* < 0.05, (**Figure 2I**)). To further distinguish between ICB-sensitive and ICB-resistant patients, we employed the TIDE algorithm, followed by BayesPrim deconvolution to analyze macrophage composition in the EMTAB3267 (**Figure S3B-D**) and TCGA-KIRC datasets (**Figure S3E-G**). The results revealed that non-responders demonstrated higher infiltration of APOE^+^ macrophages compared to responders (all *P* < 0.05). The Kappa consistency test indicated a favorable classification consistency among observers (all *P* < 0.05), suggesting the reliability of the segregated macrophages classification. Collectively, the infiltration of APOE^+^ macrophages, characterized by elevated lipid metabolism, is a strong predictor of poor immunotherapy efficacy in ccRCC.

### Upregulated CEBPD in APOE^+^ macrophages correlates with poor response to ICB therapy

**Figure 3:**
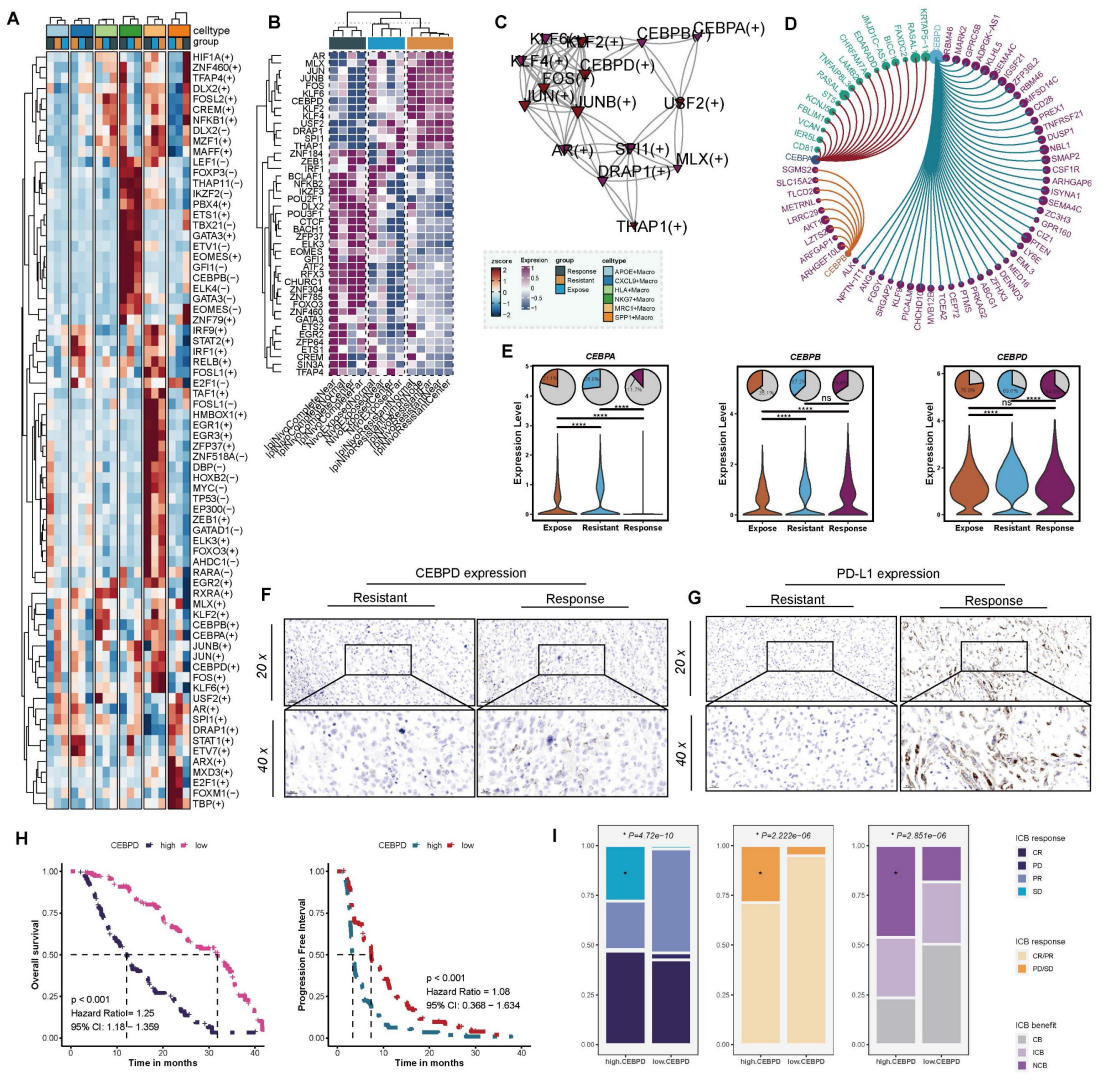
** Network of regulon-target gene interactions.** (**A**) Different regulon activities across different macrophages among the three groups. (**B**) Comparative analysis of positively regulated regulons in different tumor regions. (**C**) Network of directly activated regulons and their interactions. (**D**) CCEBPA, CEBPB, and CEBPD regulons and their top associated target genes. (**E**) Differential expression of CEBPA, CEBPB, and CEBPD across ICB groups. (**F**) Comparison of CEBPD expression between ICB-resistant and ICB-responsive patients. (**G**) Differential expression of PD-L1 between ICB-resistant and ICB-responsive patients. (**H**) Kaplan-Meier survival curves illustrating overall survival and progression-free survival stratified by CEBPD expression, using the median CEBPD value as the cutoff. (**I**) Analysis of ICB response rates based on high versus low CEBPD expression levels. CR: complete response; PR: partial response; PD: progressive disease; SD: stable disease.

To further elucidate the mechanisms by which APOE^+^ macrophages impair the efficacy of ICB therapy, we constructed a regulon-target-function network. Based on the SCENIC analysis, we identified differences in regulon activities among various macrophage populations across the ICB-responsive, ICB-exposed, and ICB-resistant groups (**Figure S4A**). Notably, APOE^+^ macrophages in resistant patients exhibited higher levels of *AR, MLX, JUN, JUNB, FOX, KLF6, CEBPA, KLF2, KLF4, CEBPB, CEBPD, USF2, SPI1*, and *DRAP1* (**Figure 3A**). Compared to other tumors, these regulons also exhibited higher expression levels in the proximal, distal, and central regions of ICB-resistant tumors, as well as in adjacent tissues. These results suggested that these regulons are potential hallmark features of ICB-resistant tumors and warrant further research (**Figure 3B**). Furthermore, we constructed directly positive regulon network specific to APOE^+^ macrophages (**Figure 3C**) to identify corresponding target genes associated with each regulon (**Figure 3D, Figure S4C-E**). Interestingly, *CEBPD* was particularly notable for directly regulating multiple genes such as* ALK, ANG, FGGY, KLF99*, and *TCEA2*. These targeted genes were enriched in immune-suppressive signaling pathways, including the negative regulation of focal adhesion assembly, negative regulation of chemotaxis, negative regulation of cell-substrate junction organization, negative regulation of B cell proliferation, and PD-L1 expression and PD-1 checkpoint pathway in cancer (**Figure S4F**).

Secretion of chemokine are crucial in recruiting T cells, APOE^+^ macrophage in ICB-resistant group showed relatively lower chemokine transcriptional activity, such as CXCL1, CXCL2, CCL4, CCL5, CCL7, CCL13, and CCL18(**Figure S4G**), among which CCL4 (*P* < 0.001) and CCL5(*P* < 0.001) (**Figure S4H**) were tightly related to CD8^+^ T cells 56. Evidence above suggested that activation of these pathways by *CEBPD* in APOE^+^ macrophages lead to the suppression of effector immune cell function by restricting the migration and activity of effector T cell and NK cells, suppressing B cell proliferation, and enhancing the PD-L1/PD-1 pathway, thereby reducing the efficacy of ICB therapy. To further asses the suitability of CEBPD as a biomarker, we systematically evaluated its expression and prognostic correlation among different ICB samples. Notably, its expression significantly increased in APOE^+^ macrophages within the ICB-resistant patients (**Figure 3E**). Furthermore, to validate these findings, we further compared the expression of *CEBPD* (**Figure 3F**) and PD-L1 (**Figure 3G**) among resistant and response patients based on IHC analysis, and the results showed that CEBPD significantly upregulated in resistant patients. Further validation was performed across two independent ICB therapy cohorts (GSE67501 cohort, N = 11; Miao et al. cohort, N = 16) revealed a trend of higher CEBPD expression in non-responders compared to responders, although the difference did not reach statistical significance (**Figure S4I**).

In a real-world cohort collected at our center, the FU-ICI cohort, high CEBPD expression related to shorter OS (*P* < 0.001, HR = 1.25, 95%CI: 1.18-1.359) and PFS (*P* < 0.001, HR = 1.08, 95%CI: 0.368-1.634) (**Figure 3H**). Patients with high CEBPD expression occurred more SD and PD events, and overall, derived less benefit from ICB strategy (all *P* < 0.05, **Figure 3I**). Taken together, these results suggest that CEBPD-driven immune suppression in APOE^+^ macrophages contribute to ICB resistance in ccRCC, highlighting *CEBPD* as a potential therapeutic target to overcome immunotherapy resistance.

### Heterogeneous epithelial populations show distinct preferences in ICB-resistant groups

**Figure 4:**
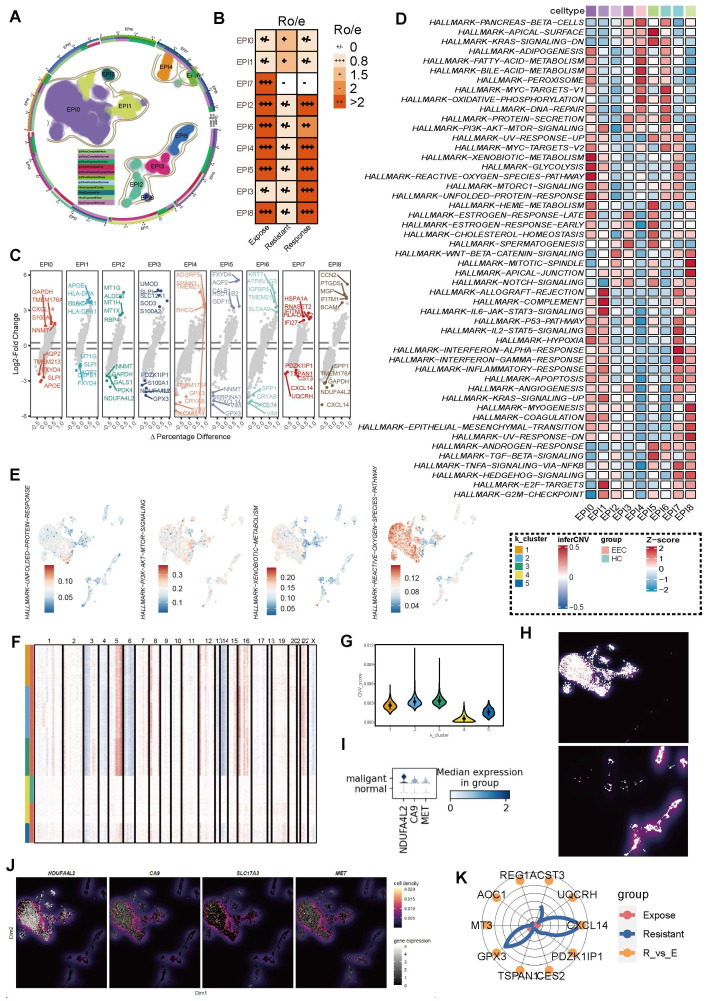
** Epithelial heterogeneity across ICB groups.** (**A**) UMAP visualization of nine epithelial cell populations. (**B**) Tissue preference of each epithelial subset, assessed using the Ro/e index. (**C**) Differential expression of upregulated and downregulated genes in each epithelial subset. (**D**) Heatmap displaying Gene Set Enrichment Analysis (GSEA) results based on the HALLMARK database. (**E**) UMAP showing the HALLMARK pathways with high activation in the EPI0 subset. (**F**) Semi-supervised clustering heatmap based on copy number variation (CNV) scores, identifying five distinct clusters. (**G**) Comparison of CNV scores across the five clusters. (**H**) Facet plot illustrating the distribution of tumor and normal cells. (**I**) Differential expression of NDUFA4L2, CA9, and MET between malignant and normal epithelial subsets, stratified by CNV scores. (**J**) Density plots showing the expression of NDUFA4L2, CA9, SLC17A3, and MET in the UMAP space. (**K**) Top ten differentially expressed genes (DEGs) in malignant epithelial cells between ICB-resistant and ICB-exposed patients.

Given the pivotal role of the TME in shaping immune responses, we next focused on the heterogeneity of epithelial populations to explore how they might contribute to ICB resistance. We identified nine distinct epithelial cell clusters across all samples (**Figure 4A**), with cluster EP0 and EP1 demonstrating a specifically stronger association with ICB-resistant groups (**Figure 4B**). Each of these epithelial clusters exhibited unique transcriptomic profiles, with differential enrichment into various HALLMARK pathways (**Figure 4C**). Specifically, EPI0 was characterized by elevated adipogenesis, xenobiotic metabolism, glycolysis metabolism, hypoxia, MTORC1 signaling and decreased Wnt/β-catenin signaling, while EPI1 was associated with allograft rejection, complement, IL6-JAK/STAT3 signaling and cell cycle regulation, such as E2F targets and G2M checkpoint (**Figure 4D-E**).

To further distinguish malignant epithelial cells in ccRCC, we inferred somatic large-scale chromosomal CNV and calculated the CNV score for each epithelial cluster, using normal epithelial cells as a reference. After obtaining the CNV score of each cell, we re-clustered all tested epithelial cells into five categories based on the scores (**Figure 4F**). Clusters 1, 2, and 3 exhibited significantly higher CNV scores compared to the other two groups (**Figure 4G-H**), thereby classifying them as tumor cells. This classification was further validated by the elevated expression of known ccRCC markers, including NDFA4L2, CA9, and MET, which were highly expressed in the malignant epithelial cells identified by CNV analysis, consistent with previous studies 57 (**Figure 4I-J**).

Additionally, we compared the gene expression profiles of tumor cells between the ICB-resistant and ICB-expose groups. Notably, genes such as *CST3*, *GPX3*, and *CXCL14* were significantly elevated in the ICB-resistant group, suggesting that these genes may serve as potential biomarkers for identifying ICB resistance in ccRCC (**Figure 4K**). Overall, these findings highlight the heterogeneity of epithelial populations in ccRCC and suggest that specific transcriptomic and genomic features, particularly in EP0 and EP1, may contribute to ICB resistance.

### Dissecting T cell subsets and their implications in ICB-resistant groups

**Figure 5:**
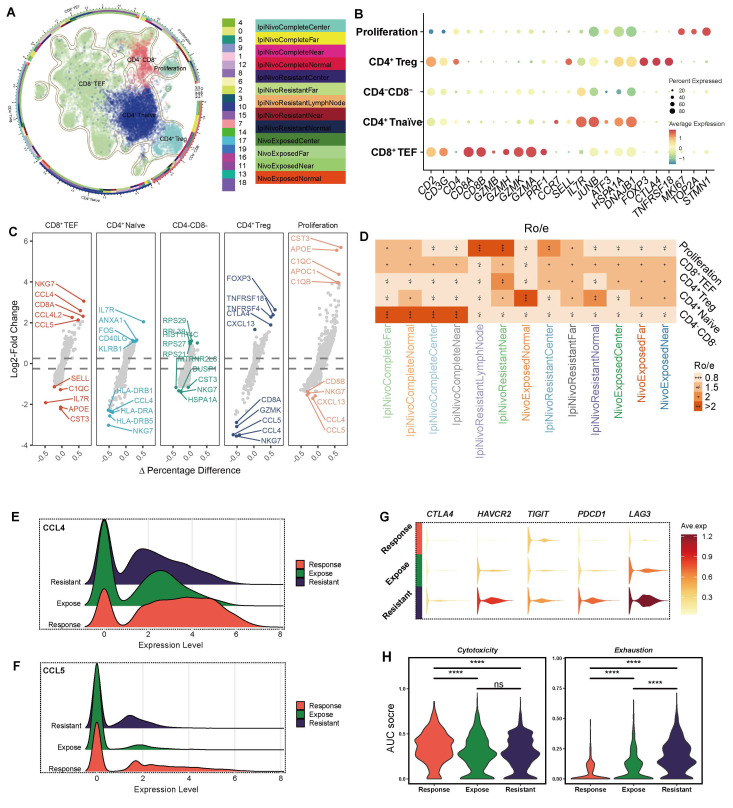
** T cell subset characterization and functional implications across ICB groups.** (**A**) UMAP visualization of T subset cells. (**B**) Dot plot displaying the signature gene expression profiles of various T cell types. (**C**) Differentially expressed genes (DEGs) among the five T cell subsets. (**D**) Tissue distribution of the five T cell subsets across different samples. (**E**) Comparison of CCL4 and CCL5 expression among the three groups. (**G**) Differential expression of exhaustion markers (CTLA4, HAVCR2, TIGIT, PDCD1, and LAG3) across the ICB groups. (**H**) CD8^+^ T effector cell status among the three ICB groups was assessed using AUCell analysis, highlighting two key features: cytotoxicity and exhaustion * represent *P* < 0.01; ** represent *P* < 0.001; *** represent *P* < 0.05.

Building on our previous findings that elucidated the clinical relevance of APOE^+^ macrophages and their impact on immunotherapy outcomes, we now turn our attention to the role of T cell dysfunction and its contribution to resistance mechanisms. We performed a detailed analysis of T cell subsets (**Figure 5A**). Using signature genes (**Figure 5B**) 58 and DEGs (**Figure 5C**), we annotated each T cell subset with precision. CD4^+^ regulatory T cells (Tregs) were characterized by high expression of CTLA4 and FOXP3, while CD8^+^ effector T cells (TEFs) were defined by the expression of GZMB and GZMH. Additionally, CD4^+^ naïve T cells were characterized by high expression of CCR7 and IL7R.

Notably, CD4^+^ Tregs were predominantly localized to the peritumoral regions in ICB-resistant samples, suggesting their involvement in therapy failure (**Figure 5D**). Through DEGs analysis, we observed that CD8^+^ TEF exhibited higher expression levels of CCL4 (**Figure 5E**) and CCL5 (**Figure 5F**), both of which facilitate the recruitment of macrophages, dendritic cells, and other T cells into the tumor microenvironment. Notably, among the three groups analyzed, the expression of CCL4 and CCL5 was relatively low in the ICB-resistant group, indicating a compromised functional capacity of CD8^+^ TEFs in these tumors. This diminished chemokine secretion may hinder their ability to orchestrate an effective anti-tumor immune response.

Further functional assays revealed that ICB-resistant tumors expressed higher levels of exhaustion markers, including CTLA4, HAVCR2, TIGIT, PDCD1, and LAG3 (**Figure 5G**), all of which are strongly associated with immune exhaustion 59. To systematically assess the functional status of CD8^+^ TEF cells, we utilized the AUCell score across the different groups (**Figure 5H**). The cytotoxicity score was highest in the ICB-response group (*P* < 0.001), whereas the exhaustion score peaked in the ICB-resistant group (*P* < 0.001), corroborating our earlier findings. Collectively, ICB-resistant tumor exhibits an exhausted CD8^+^ TEF phenotype and demonstrate a reduced capacity for chemokine secretion, potentially influenced by APOE+ macrophages or tumor cell reprogramming. This immune dysfunction underscores a critical barrier to effective immunotherapy in these tumors.

### Tumor-derived SPP1 signaling promotes APOE^+^ macrophage polarization and TGF-β secretion to establish an immunosuppressive microenvironment

**Figure 6:**
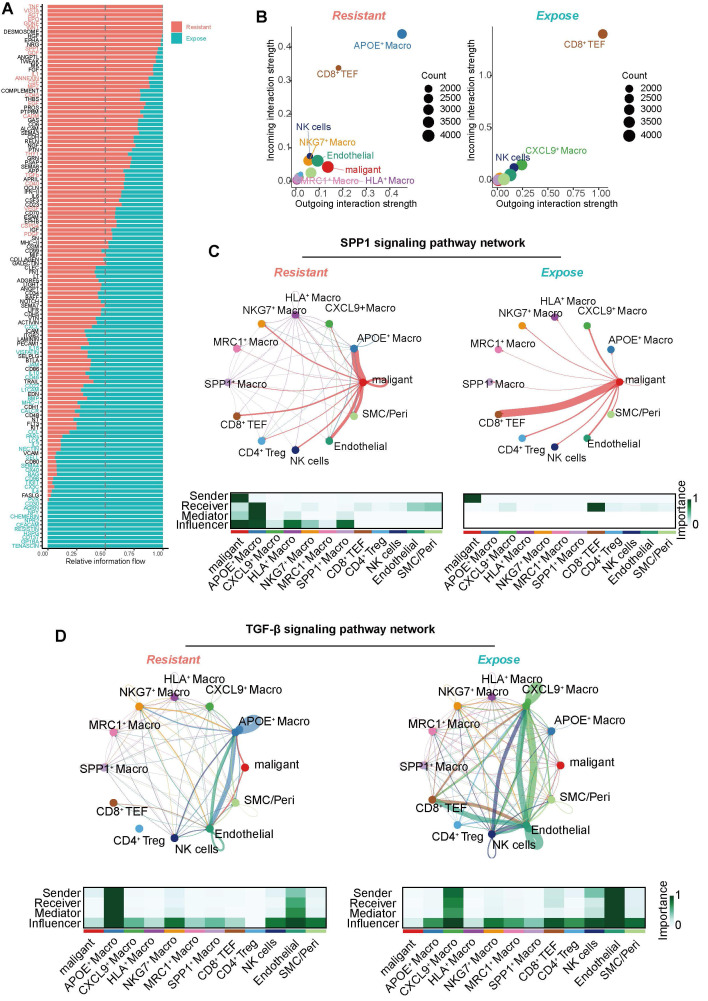
** Intercellular communication alterations in ICB resistant and exposed patients.** (**A**) Comparison of overall signaling pathway activity between ICB-resistant and ICB-exposed patients. Red indicates high enrichment in the ICB-resistant group, green indicates high activation in the ICB-exposed group, and black indicates no significant difference between the two groups. (**B**) Total incoming and outgoing signaling strength for different cell types across ICB groups. (**C**) Comparison of the SPP1 signaling network. Ligand-receptor interaction weights in ICB-resistant patients (top left) and ICB-exposed patients (top right). The heatmap displays the dominant senders, receivers, mediators, and influencers in SPP1 signaling, based on network centrality scores. (**D**) Comparison of the TGF-β signaling network. Ligand-receptor interaction weights in ICB-resistant patients (top left) and ICB-exposed patients (top right). The heatmap displays the dominant senders, receivers, mediators, and influencers in TGF-β signaling, based on network centrality scores.

Furthermore, we conducted a comprehensive Cell-to-Cell Communication analysis to assess signaling dynamics within the tumor immune microenvironment. Distinct cell-to-cell communication patterns were revealed between the ICB-exposed and ICB-resistant groups (**Figure 6A**). The ICB-resistant group displayed stronger and more active signaling pathways, including *TNF, VISTA, AGT, EPO, WNT, SPP1, GDF, IL1, CSF, CD45, EFG*, and TGF-β signaling. Among all cell types, APOE^+^ macrophages exhibited the most prominent incoming and outgoing signaling activities (**Figure 6B**).

In the SPP1 signaling pathway network, malignant epithelial emerged as the primary signal sender, while APOE^+^ macrophages acted as the predominant receiver, medicator and influencer within the ICB-resistant group (**Figure 6C**). Although macrophage-derived SPP1 is well-documented to promote macrophage polarization toward an M2-like, immunosuppressive phenotype, the role of epithelial-derived SPP1 in this process has been less explored 60. Our results suggest that tumor-derived SPP1 may similarly promote the polarization of macrophages into an immunosuppressive state, indicating that SPP1 could be a potential therapeutic target in ICB-resistant tumors. Moreover, we also found APOE^+^ macrophages were the largest source of TGF-β signaling in ICB-resistant group (**Figure 6D**), which is known to play a central role in shaping an immunosuppressive microenvironment, further contributing to immune evasion and tumor progression 61. Therefore, these findings indicate that tumor-derived SPP1 signaling drives the polarization of APOE^+^ macrophages, which subsequently secrete TGF-β to establish an immunosuppressive microenvironment, contributing to the failure of immunotherapy in ICB-resistant ccRCC.

### Spatially segregated APOE^+^ macrophages at the tumor border mediate immunosuppressive communication and structural organization

**Figure 7:**
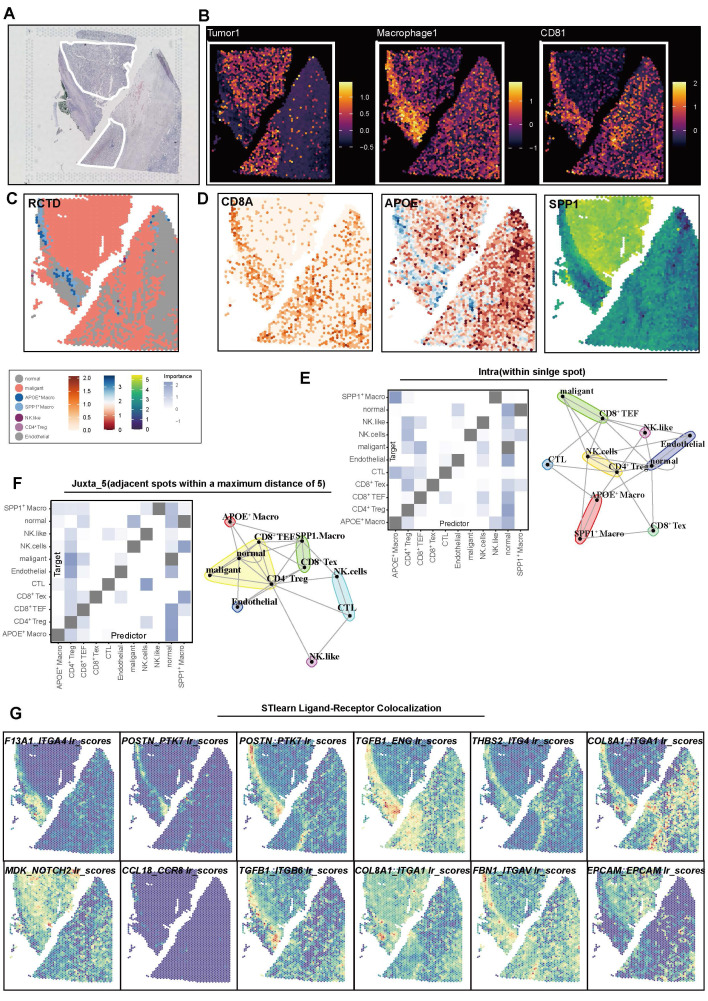
** Spatial colocalization and intercellular communication analysis.** (**A**) Hematoxylin and eosin (H&E) staining. (**B**) Spatial plot showing feature scores for tumors, macrophages, and CD8^+^ T cells. (**C**) Robust Cell Type Decomposition (RCTD) deconvolution revealing the cell type composition at each spatial spot. (**D**) Spatial plot showing the expression of CD8A, APOE, and SPP1 across spatial spots. (**E**) Heatmap illustrating intra-context spatial dependencies of cell types based on RCTD results (left), and a network plot showing cell-to-cell interactions within spatial spots (right). (**F**) Heatmap illustrating juxta-context spatial dependencies of cell types based on RCTD results (left), and a network plot showing cell-to-cell interactions between spatial spots (right). (**G**) Spatial plot showing the colocalization of ligand-receptor pairs based on stLearn analysis.

Building on our previous findings regarding the spatial arrangement of immune cell populations, we next focused on the localization and functional role of APOE^+^ macrophages at the tumor border. We initially confirmed the tumor regions based on morphological features (**Figure 7A**). By utilizing the malignant markers (*NDFA4L2, CA9, MET*), macrophages (*APOC1, C1QA, C1QB, CD14, CD16, CD68, CD163*), and CD8^+^ T cell (*CD2, CD3D, CD3E, CD8A, CD8B*), we calculated corresponding scores to map the relative spatial position of these cells (**Figure 7B**). To further delineate the spatial distribution of celltypes within the TME, we applied the RCTD method (**Figure 7C**), which deconvoluted scRNA sequencing data into spatial data, allowing the inference of primary cell types at each spatial location. Expression of key molecules, such as APOE, SPP1, and CD8A, validated the deconvolution results (**Figure 7D**).

These findings showed that APOE^+^ macrophages are predominantly distributed along the tumor border regions, forming a physical immune barrier between CD8^+^ T cells and tumor cells. In the MISTy analysis, APOE^+^ macrophages emerged as central mediators of cell-to-cell communication within the TME. The intra- and jux-context interaction networks demonstrate that APOE^+^ macrophages engage in extensive interactions with other immune cells, such as CD8^+^ Tex cells, CD4^+^ Tregs, and malignant cells (**Figure 7E-F**). These macrophages exbited higher predicted communication importance scores, underscoring their critical role in shaping an immune-suppressive microenvironment niche.

Further investigation using Stlearn analysis explored spatial patterns of ligand-receptor interactions involving APOE^+^ macrophages. Elevated interaction scores were observed for key ligand-receptor pairs, such as ITGAL_ITGAL, COL3A1_ITGA1, CCL18_CCR1, and TGF-β signaling, particularly in regions with high APOE^+^ macrophage activity. These interactions were concentrated in regions of immune cell infiltration and structural support, suggesting that APOE^+^ macrophages help establish immune-suppressive niches. Notably, the enrichment of collagen and integrin interactions (e.g., COL1A1_ITGA1) further indicates that APOE^+^ macrophages organize the tumor stroma elements to facilitate tumor progression and immune evasion (**Figure 7G, Figure S5**).

### Upregulated SPP1 promotes the proliferation and migration of 786-O and 769-P cells

**Figure 8:**
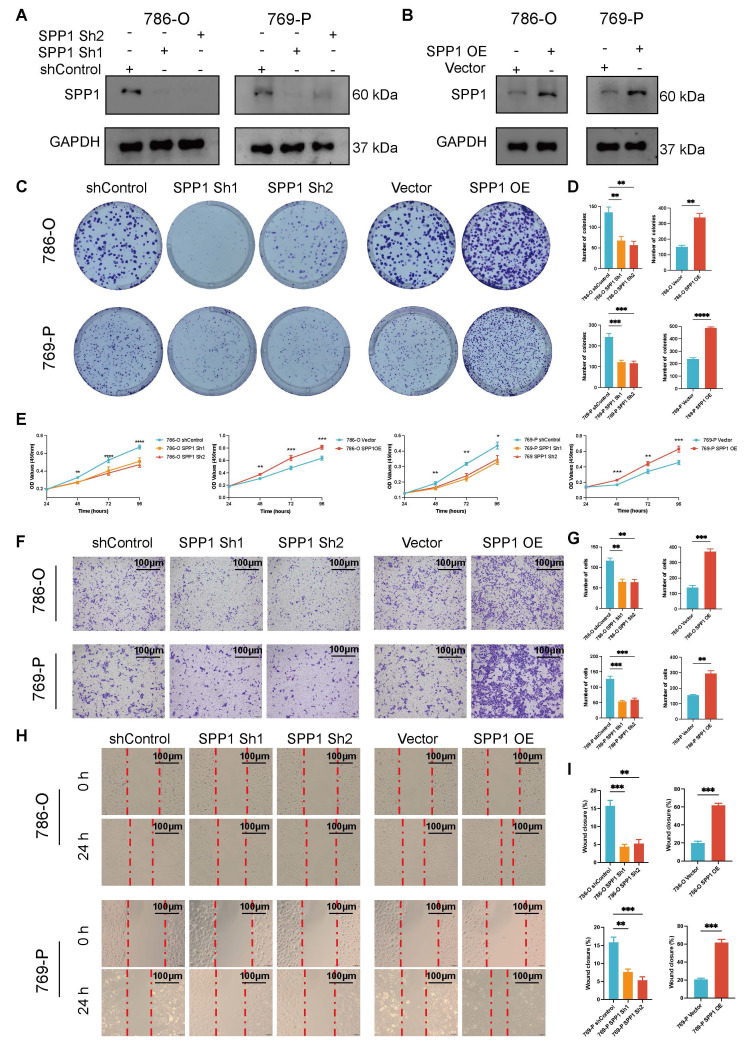
** Upregulated SPP1 expression promotes the proliferation and migration of 786-O and 769-P cells.** (**A**) The levels of SPP1 expression in 786-O and 769-P cell lines after SPP1 knockdown are shown through Western blot analysis, with GAPDH used as the internal control. (**B**) The Western blot analysis indicates the expression of SPP1 in 786-O and 769-P cell lines following its overexpression. (**C**) Representative images from colony formation assays in 786-O and 769-P cells, featuring both SPP1 knockdown (Sh1 and Sh2) and overexpression, are compared to their respective controls. (**D**) The quantification and comparison of colony counts are depicted (* represent *P* < 0.05; ** represent *P* < 0.01; *** represent *P* < 0.001). (**E**) The CCK-8 assay for proliferation was conducted to investigate how varying levels of SPP1 expression impact cell growth. (**F**) Transwell migration assay was performed to assess the ability of renal cancer cells to migrate after SPP1 knockdown or overexpression. (**G**) The quantity of cells that migrated was measured. (**H**) Representative images show SPP1 knockdown and overexpression during the wound healing assays at 0 hours and 24 hours. (**I**) A quantitative assessment of the percentage of wound closure is presented.

Compared to the shControl group, the SPP1 protein levels in the ccRCC cell lines (786-O and 769-P) significantly decreased following shRNA knockdown (**Figure 8A**). Concurrently, low expression of SPP1 in the Vector control group and high in SPP1 OE group indicated successful model construction (**Figure 8B**). Both in the 786-O and 769-P cell line, significant reduction in the number of clones in the culture dishes for the SPP1 Sh1 and SPP1 Sh2 groups, and SPP1 OE significantly enhanced the ability of cell clone formation (all *P* < 0.05, **Figure 8C-D**). After four days, SPP1 Sh1 and SPP1 Sh2 groups showed a marked decrease in their proliferation capabilities, while the SPP1 OE group displayed a notable enhancement in proliferation (all *P* < 0.05, **Figure 8E**).

In terms of the impact of SPP1 on migration ability of ccRCC, the SPP1 Sh1 and Sh2 groups demonstrated a significant decrease in the number of transmembrane cells compared with shControl, while the SPP1 OE group (*P* < 0.05) exhibited the highest number of transmembrane cells, suggesting that SPP1 overexpression significantly enhanced cell migration ability (all *P* < 0.05, **Figure 8F-G**). Compared to shControl groups, the Sh1 and Sh2 groups displayed a significantly reduced migratory ability, with the degree of scratch healing being lower than that of the control group, as the scratch remained evident after 24 h. The SPP1 OE group showed a significant improvement in migration ability, with the highest degree of scratch healing. After 24 h, the scratch area was almost completely filled with cells (all *P* < 0.05, **Figure 8H-I**). Taken together, these experiments comprehensively confirmed that the expression level of the SPP1 protein is closely related to the malignant biological behavior of ccRCC cells.

### SPP1 overexpression drives APOE^+^ M2-like macrophage polarization, promoting tumor progression and immunosuppressive TME formation

**Figure 9:**
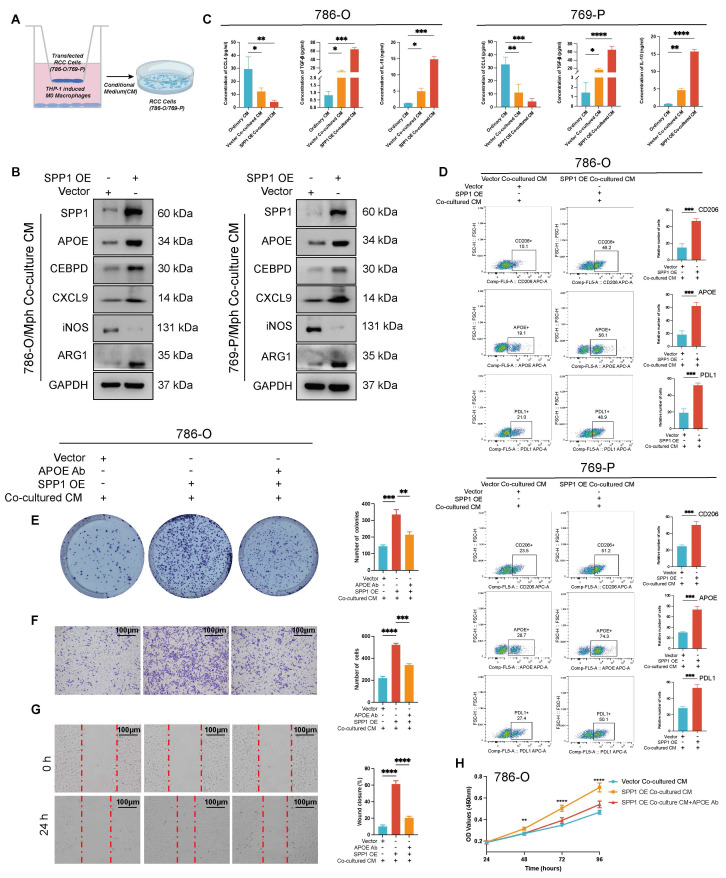
** SPP1 overexpression drives APOE+ M2-like macrophage polarization, promoting tumor progression and immunosuppressive TME formation.** (**A**) The schematic diagram illustrates the co-culture system in which THP-1-derived macrophages are cultured alongside the CM from SPP1-overexpressing renal cancer cells (co-culture CM) to investigate macrophage polarization and its effects on tumor progression, including Ordinary CM, Vector Co-cultured CM, and SPP1 OE Co-cultured CM. (**B**) Western blot analysis demonstrated that after co-culturing SPP1-overexpressing 786-O cells or 769-P with THP-1-derived macrophages, the protein levels of SPP1, iNOS (M1 marker), APOE, CEBPD, CXCL9, and ARG1 (M2 marker) were assessed, with GAPDH serving as the internal reference. (**C**) ELISA results indicated the secretion levels of CCL4, TGF-β, and IL-10 in the conditioned medium from 786-O and 769-P cells (* represent *P* < 0.05; ** represent *P* < 0.01; *** represent *P* < 0.001). (**D**) Flow cytometry analysis was conducted to evaluate the expression of CD206, APOE, and PD-L1 in macrophages among Vector Co-cultured CM and SPP1 OE Co-cultured CM. (**E**) A colony formation assay was performed to assess the impact of co-culture CM and APOE neutralization on the cloning ability of 786-O cells. (**F**) Transwell migration assays analysis showed the different migration ability of 786-O cells. (**G**) Evaluation of the effects of co-culturing CM and APOE neutralization on cell migration by wound healing analysis. (**H**) Assess the impact of co-culturing CM and APOE neutralization on tumor cell proliferation by CCK-8 proliferation analysis.

In the following analysis, as showed in **Figure 9A**, ccRCC cells (786-O and 769-P) transfected with SPP1 overexpression or control vectors were co-cultured with THP-1 derived macrophages, followed by CM collection for subsequent analysis. Next, we employed WB analysis to elucidate the impact of SPP1 OE in renal cancer cells on macrophage. The results indicated that the SPP1 OE Co-cultured CM group significantly increased the protein levels of SPP1, APOE, ARG1 and CEBPD.

Concurrently, the expression of iNOS was notably reduced, while changes in CXCL9 expressions were minimal. This suggested that SPP1 OE induced macrophage polarization towards APOE^+^ macrophages (**Figure 9B**, **Figure S6A**), alongside an increase in CEBPD expression. Notably, CCL4 levels significantly decreased after co-culture, particularly in the SPP1 OE Co-cultured CM group (*P* < 0.01). Furthermore, TGF-β and IL-10 levels were significantly elevated (*P* < 0.001) (**Figure 9C**). This finding aligns with our previous hypothesis. Additionally, by assessing the expression changes of macrophage polarization markers CD206, APOE, and PDL1 using flow cytometry, we observed that in both 786-O and 769-P cells, the SPP1 OE Co-cultured CM demonstrated a significant increase in CD206, APOE, and PDL1 compared to the ordinary CM (*P* < 0.001, *P* < 0.0001) (**Figure 9D**). This evidence suggested that high SPP1 CM drive macrophage polarization toward an APOE^+^ M2-like Macrophage phenotype, which was consistent with above.

Furthermore, we utilized the Vector Co-cultured CM, the SPP1 OE Co-cultured CM group, and the SPP1 OE Co-culture CM+APOE Ab group to investigate the malignant progression of renal cancer cells in the presence of APOE^+^ M2-like macrophages. The results of the plate cloning formation demonstrated that the SPP1 OE Co-cultured CM group significantly increased the number of tumor cell clones. After the addition of the anti-APOE antibody, the clonal formation ability of the tumor cells was significantly reduced (all *P* < 0.05,** Figure 9E**, **Figure S6A**). Transwell migration experiment confirmed that the number of migrated cells in the SPP1 OE Co-cultured CM group was significantly higher than that in the Vector Co-cultured CM; after adding the anti-APOE antibody, the number of migrated cells decreased significantly (all *P* < 0.05, **Figure 9F**, **Figure S6B**). The SPP1 OE Co-cultured CM group showed the highest wound closure rate, indicating that APOE^+^ macrophage plays a crucial role in promoting migration (all *P* < 0.05, **Figure 9G**, **Figure S6C**). The CCK-8 cell proliferation analysis revealed that the SPP1 OE Co-cultured CM group significantly promoted cell proliferation, as evidenced by a substantial increase in the OD value (*P* < 0.05, **Figure 9H**, **Figure S6D**). Following the addition of the anti-APOE antibody, the cell proliferation curve exhibited a significant decrease.

Collectively, the experimental results align with previous findings from single-cell spatial multi-omics studies. Tumor-derived SPP1 promotes the polarization of macrophages into APOE^+^ M2-like macrophages, upregulates CEBPD, inhibits the release of chemokines, enhances the activation of the PD-L1 pathway, and modulates immune suppressive signals such as TGF-β and IL-10, thereby shaping an immunosuppressive microenvironment.

## Discussion

Malignant renal cancer is characterized by high invasiveness, with advanced renal cancer being particularly prone to metastasis. It is resistant to conventional chemotherapy and radiotherapy, making treatment very challenging once metastasis occurs 62, 63. Therefore, effective early intervention is crucial in renal cancer treatment. Currently, immunotherapy has become the fourth treatment modality alongside surgery, radiotherapy, and chemotherapy, and is increasingly applied in clinical settings, especially for patients with metastatic renal cancer 64, 65. Nonetheless, the mechanisms that contribute to resistance are still not well comprehended. We comprehensively analyzed the scRNA transcriptomes of both cancer and immune cells in patients with metastatic RCC, both prior to and following ICB treatment.

Macrophage-derived APOE tightly correlated to shorter progression of recurrence and poor prognosis of renal cancer 66. APOE expressed by macrophages inhibits inflammation by transmitting anti-inflammatory signals via extracellular vesicles. Wild-type macrophage vesicles increase levels of apoE and miR-146a-5p, reducing NF-κB signaling and enhancing fatty acid oxidation and oxidative phosphorylation, thus promoting an anti-inflammatory metabolism. In contrast, apoE-deficient vesicles promote NF-κB activity and oxidative stress 67. Liu *et al.* 29 primarily explored the potential role of APOE^+^ macrophage in ICB therapy among pan-cancer analysis, and the infiltration might be related CD8^+^T exhausted cells and to ICB therapy failure in triple negative breast cancer (TNBC). In this study, we identified the role of APOE^+^ macrophage infiltration in ICB failure of RCC and comprehensively explore the molecular mechanisms based on combination of single-cell and spatial transcriptome multi-omics.

In the results, significantly elevated APOE^+^ macrophage infiltrated in ICB-resistant TME compared to ICB response and expose. More than 80% of macrophage in the ICB-resistant group were APOE^+^ macrophage, while the proportion was less than 10% in the other two subgroups. In mIF analysis, we also more infiltration of M2 macrophage in ICB-resistant patient with elevated APOE expression, indicating the anti-inflammatory function. This evidence indicated the role of APOE^+^ macrophage in ICB failure. In addition, we found APOE^+^ macrophage might be the terminal cells which indicated tumor might promote macrophage to polarized in the direction of APOE^+^ macrophage under some extent signaling. To solve this, we established Regulons-targets network and performed enrichment analysis. Among these regulons, CEBPD might be a key molecule for suppressing immunotherapy of APOE^+^ macrophage. CEBPD target genes are heavily enriched in immunosuppression-related singling, inhibits the production of chemokines and cell adhesion thereby preventing T cells from infiltrating the local milieus and creating a relatively immune microenvironment. In addition, we found elevated CEBPD could upregulate PD-L1 expression in RCC to suppress T cell mediated anti-tumor response, contributing to tumor escape. Coincidently, Zhao et al. 68 also found the CEBPD can activate expression of VAMP3 in TNBC, which further increase the secretion of PD-L1 in extracellular vesicles, thereby enhancing the chemoresistance of TNBC. In IHC analysis, increased expression of CEBPD also be found in Resistance tumor, as well as the results in two external cohort.

SPP1 is widely expressed in many immunocytes and tumor cells, and has a prominent role in tumor development and immune response. In glioma, SPP1 plays as a chemokine that drives the infiltration of TAM to suppress anti-tumor response 69. In present studies, SPP1 expression contributed to IO-TKI resistance and shorter progression-free time in RCC. Similarly, our results validated that upregulated SPP1 enhances the proliferation, clonal formation and migration abilities of renal cancel cells, suggesting the essential role of SPP1 in promoting tumor progression. However, the mechanism for immune evasion centered on SPP1 in RCC still unclear 70. In this study, we found that ICB-resistant RCC presented significant activation of SPP1, and directly target APOE^+^ macrophage. Co-culture experiments revealed that the increased expression of SPP1 in renal cancer cells led to a marked polarization of APOE^+^ M2-like macrophages, elevated the levels of immunosuppressive markers like APOE and CEBPD. This polarization fostered an immunosuppressive microenvironment that facilitated tumor progression, as indicated by heightened tumor cell proliferation, migration, and colony formation. Importantly, the use of anti-APOE antibodies significantly counteracted these effects. When APOE^+^ macrophage as singling sender, many immunosuppressive singling was deciphered, where TGF-β attracting much attention. TGF-β was famous for its central role in formulating suppressive TME 71, which were also upregulation in ICB-resistant RCC. In resistant patients, APOE^+^ macrophage secreted prominent TGF-β singling to inhibit T cell activation. In addition, we found APOE^+^ macrophage gather accumulate around tumor borders, as well as abundant TGF-β signaling. Together form a barrier at the tumor boundary inhibiting the infiltration and activating of T cells, creating good conditions for the growth and progression of malignant cells. Meanwhile, hindering the effective infiltration of T cells, thereby inhibiting the efficacy of ICB therapy.

This research has revealed a variety of innovative therapeutic targets with potential applications in the treatment of cancer, along with insights into their mechanisms of action. The Anushka team has shown through their studies that the targeting and removal of important elements such as CD73, CSF1, or SPP1 secreted by quiescent metastatic cancer cells can significantly disrupt the establishment of the immunosuppressive TME. This disruption may resensitize quiescent metastatic tumors that were earlier unresponsive to ICB therapy 72. As described above, Liu et al. validated that combining the APOE inhibitor COG 133 TFA with anti-PD-1 therapy yields a marked synergistic anti-tumor response in TNBC, indicating that targeting APOE can significantly enhance the effectiveness of immune checkpoint inhibitors 29. Comprehensive mechanistic investigations have demonstrated that APOE^+^ macrophages contribute to a complex immunosuppressive network by enhancing the expression of the transcription factor CEBPD along with its downstream target genes, reducing the release of chemokines, which in turn hinders the infiltration of CD8+ T cells. Furthermore, it diminishes the phagocytic abilities of macrophages and strengthens the immunosuppressive microenvironment via PTX3 and IL-10 73, 74. These observations lay a theoretical framework for the advancement of inhibitors targeting CEBPD in conjunction with immunotherapy. Notably, TGF-β, the principal effector molecule modulated by APOE^+^ macrophages, has been recognized as a key player in immunosuppression. The bispecific antibody LBL-015, which combines anti-PD-1 monoclonal antibodies with the extracellular domain of TGF-βRII, was created stemming from this research and has shown promising safety profiles in Phase I clinical trials. Out of 25 patients enrolled with advanced solid tumors, one individual with renal cancer experienced a partial response lasting more than 28 weeks (NCT05107011) 75.

This study presents a few limitations. To begin with, the data utilized in this research came from a limited group of patients, which could limit how broadly the results can be applied. Additionally, the single-cell RNA sequencing and spatial transcriptomics technologies employed present challenges in directly analyzing protein level regulation, thereby hindering a comprehensive understanding of the interactions involving APOE^+^ macrophage at the protein level. Furthermore, single-cell sequencing data are often noisy, potentially compromising the accuracy of gene expression measurements. Lastly, this study relies on static single-cell and spatial transcriptome data, which do not capture the dynamic changes occurring within the tumor microenvironment, thus limiting insights into the evolutionary trajectory of APOE^+^ macrophage before and after ICB treatment.

## Conclusion

In conclusion, our study highlights the critical role of APOE^+^ macrophages in promoting immunotherapy resistance and immunosuppressive TME in ccRCC. This study integrates single-cell spatial multi-omics a, mIF and multiple experiments to uncover the pivotal role of APOE^+^ macrophages in fostering an in ccRCC. Tumor-derived SPP1 signaling recruits and polarizes APOE^+^ macrophages to the peri-tumoral regions and tumor borders, where they secrete TGF-β, upregulated CEBPD, and triggered PD-L1 signaling, forming a physical and functional barrier between CD8^+^ T cells and malignant cells, thereby effectively dampening immune responses. Therfore, targeting the APOE^+^ macrophage population and their associated signaling pathways could represent a promising therapeutic strategy to overcome immunotherapy resistance in ccRCC, potentially improving clinical outcomes for patients.

## Supplementary Material

Supplementary figures and tables.

## Abbreviations

RCC: Renal cell carcinoma
ccRCC: Clear cell renal cell carcinoma
TME: Tumor microenvironment
PCA: Principal component analysis
PAGA: Partition-based graph abstraction
ICB: Immune checkpoint blockade
mIF: Multiplex immunofluorescence
IHC: Immunohistochemistry
APOE: Polipoprotein E
CEBPD: CCAAT enhancer binding protein delta
SPP1: Secreted phosphoprotein 1
NCCN: The national comprehensive cancer network
IMDC: The international metastatic RCC database consortium
TAMs: Tumor-associated macrophages
Tregs: Regulatory T cells
TLS: Tertiary lymphoid structures
Tex: T effector exhausted
scRNA-seq: Single-cell RNA sequencing
ENA: The European nucleotide archive
TCGA: The cancer genome atlas
TIDE: The tumor immune dysfunction and exclusion
TPM: Transcripts per million
OS: Overall survival
PFS: Progression free interval
TFs: Transcription factors
Regulon: Transcription factor
CNV: Copy number variation
HMM: Default hidden Markov model
L-R: Ligand to receptor
ST: Spatial transcriptomics
RCTD: Robust cell type decomposition
FFPE: Formalin-fixed, paraffin-embedded
DAPI: 4',6-diamidino-2-phenylindole
CK: Cytokeratin
UMAP: Uniform manifold approximation and projection
SMC/peri: Muscle/pericytes
CB: Clinical benefit
NCB: No clinical benefit
PD: Progressive disease
SD: Stable disease
CR: Complete response
PR: Partial response
DEGs: Differentially expressed genes
LA-TAMs: Lipid-associated tumor-associated macrophages
TEFs: Effector T cells
TNBC: Triple negative breast cancer
shRNA: Short hairpin RNA
OE: OverExpression
CM: Conditioned medium
WB: Western blot

## References

[1] Siegel RL, Miller KD, Jemal A (2019). Cancer statistics, 2019. CA Cancer J Clin.

[2] Hsieh JJ, Purdue MP, Signoretti S, Swanton C, Albiges L, Schmidinger M (2017). Renal cell carcinoma. Nat Rev Dis Primers.

[3] Meng J, Jiang A, Lu X, Gu D, Ge Q, Bai S (2023). Multiomics characterization and verification of clear cell renal cell carcinoma molecular subtypes to guide precise chemotherapy and immunotherapy. Imeta.

[4] Chakiryan NH, Jiang DD, Gillis KA, Green E, Hajiran A, Hugar L (2021). Real-World Survival Outcomes Associated With First-Line Immunotherapy, Targeted Therapy, and Combination Therapy for Metastatic Clear Cell Renal Cell Carcinoma. JAMA Netw Open.

[5] Marona P, Górka J, Mazurek Z, Wilk W, Rys J, Majka M (2017). MCPIP1 Downregulation in Clear Cell Renal Cell Carcinoma Promotes Vascularization and Metastatic Progression. Cancer Res.

[6] Soares A, Monteiro FSM, da Trindade KM, Silva AGe, Cardoso APG, Sasse AD (2024). Advanced renal cell carcinoma management: the Latin American Cooperative Oncology Group (LACOG) and the Latin American Renal Cancer Group (LARCG) consensus update. Journal of Cancer Research and Clinical Oncology.

[7] Xu Y, Li L, Yang W, Zhang K, Zhang Z, Yu C (2023). TRAF2 promotes M2-polarized tumor-associated macrophage infiltration, angiogenesis and cancer progression by inhibiting autophagy in clear cell renal cell carcinoma. J Exp Clin Cancer Res.

[9] Raimondi A, Randon G, Sepe P, Claps M, Verzoni E, de Braud F (2019). The Evaluation of Response to Immunotherapy in Metastatic Renal Cell Carcinoma: Open Challenges in the Clinical Practice. Int J Mol Sci.

[10] Borcherding N, Vishwakarma A, Voigt AP, Bellizzi A, Kaplan J, Nepple K (2021). Mapping the immune environment in clear cell renal carcinoma by single-cell genomics. Commun Biol.

[11] Liu J, Shi Y, Zhang Y (2023). Multi-omics identification of an immunogenic cell death-related signature for clear cell renal cell carcinoma in the context of 3P medicine and based on a 101-combination machine learning computational framework. Epma j.

[12] Tumeh PC, Harview CL, Yearley JH, Shintaku IP, Taylor EJ, Robert L (2014). PD-1 blockade induces responses by inhibiting adaptive immune resistance. Nature.

[13] Qiu J, Fu Y, Liu T, Wang J, Liu Y, Zhang Z (2024). Single-cell RNA-seq reveals heterogeneity in metastatic renal cell carcinoma and effect of anti-angiogenesis therapy in the pancreas metastatic lesion. Cancer Lett.

[14] Wolf MM, Madden MZ, Arner EN, Bader JE, Ye X, Vlach L (2024). VHL loss reprograms the immune landscape to promote an inflammatory myeloid microenvironment in renal tumorigenesis. J Clin Invest.

[15] Turley SJ, Cremasco V, Astarita JL (2015). Immunological hallmarks of stromal cells in the tumour microenvironment. Nat Rev Immunol.

[16] Braun DA, Hou Y, Bakouny Z, Ficial M, Sant' Angelo M, Forman J (2020). Interplay of somatic alterations and immune infiltration modulates response to PD-1 blockade in advanced clear cell renal cell carcinoma. Nat Med.

[17] Alexandrov LB, Nik-Zainal S, Wedge DC, Aparicio SA, Behjati S, Biankin AV (2013). Signatures of mutational processes in human cancer. Nature.

[18] Turajlic S, Litchfield K, Xu H, Rosenthal R, McGranahan N, Reading JL (2017). Insertion-and-deletion-derived tumour-specific neoantigens and the immunogenic phenotype: a pan-cancer analysis. Lancet Oncol.

[19] DeNardo DG, Ruffell B (2019). Macrophages as regulators of tumour immunity and immunotherapy. Nat Rev Immunol.

[20] Anderson NR, Minutolo NG, Gill S, Klichinsky M (2021). Macrophage-Based Approaches for Cancer Immunotherapy. Cancer Res.

[21] Zheng N, Wang T, Luo Q, Liu Y, Yang J, Zhou Y (2023). M2 macrophage-derived exosomes suppress tumor intrinsic immunogenicity to confer immunotherapy resistance. Oncoimmunology.

[22] Lin H, Wei S, Hurt EM, Green MD, Zhao L, Vatan L (2018). Host expression of PD-L1 determines efficacy of PD-L1 pathway blockade-mediated tumor regression. J Clin Invest.

[23] Mantovani A, Sozzani S, Locati M, Allavena P, Sica A (2002). Macrophage polarization: tumor-associated macrophages as a paradigm for polarized M2 mononuclear phagocytes. Trends Immunol.

[24] Henze AT, Mazzone M (2016). The impact of hypoxia on tumor-associated macrophages. J Clin Invest.

[25] Kryczek I, Zou L, Rodriguez P, Zhu G, Wei S, Mottram P (2006). B7-H4 expression identifies a novel suppressive macrophage population in human ovarian carcinoma. J Exp Med.

[26] Xu W, Lu J, Liu WR, Anwaier A, Wu Y, Tian X (2023). Heterogeneity in tertiary lymphoid structures predicts distinct prognosis and immune microenvironment characterizations of clear cell renal cell carcinoma. J Immunother Cancer.

[27] Bill R, Wirapati P, Messemaker M, Roh W, Zitti B, Duval F (2023). CXCL9:SPP1 macrophage polarity identifies a network of cellular programs that control human cancers. Science.

[28] Ma RY, Black A, Qian BZ (2022). Macrophage diversity in cancer revisited in the era of single-cell omics. Trends Immunol.

[29] Liu C, Xie J, Lin B, Tian W, Wu Y, Xin S (2024). Pan-Cancer Single-Cell and Spatial-Resolved Profiling Reveals the Immunosuppressive Role of APOE+ Macrophages in Immune Checkpoint Inhibitor Therapy. Adv Sci (Weinh).

[30] Krishna C, DiNatale RG, Kuo F, Srivastava RM, Vuong L, Chowell D (2021). Single-cell sequencing links multiregional immune landscapes and tissue-resident T cells in ccRCC to tumor topology and therapy efficacy. Cancer Cell.

[31] Davidson G, Helleux A, Vano YA, Lindner V, Fattori A, Cerciat M (2023). Mesenchymal-like Tumor Cells and Myofibroblastic Cancer-Associated Fibroblasts Are Associated with Progression and Immunotherapy Response of Clear Cell Renal Cell Carcinoma. Cancer Res.

[32] Jiang P, Gu S, Pan D, Fu J, Sahu A, Hu X (2018). Signatures of T cell dysfunction and exclusion predict cancer immunotherapy response. Nat Med.

[33] Ascierto ML, McMiller TL, Berger AE, Danilova L, Anders RA, Netto GJ (2016). The Intratumoral Balance between Metabolic and Immunologic Gene Expression Is Associated with Anti-PD-1 Response in Patients with Renal Cell Carcinoma. Cancer Immunol Res.

[34] Miao D, Margolis CA, Gao W, Voss MH, Li W, Martini DJ (2018). Genomic correlates of response to immune checkpoint therapies in clear cell renal cell carcinoma. Science.

[35] Xu W, Lu J, Tian X, Ye S, Wei S, Wang J (2024). Unveiling the impact of tertiary lymphoid structures on immunotherapeutic responses of clear cell renal cell carcinoma. MedComm (2020).

[36] Hao Y, Hao S, Andersen-Nissen E, Mauck WM 3rd, Zheng S, Butler A (2021). Integrated analysis of multimodal single-cell data. Cell.

[37] McGinnis CS, Murrow LM, Gartner ZJ (2019). DoubletFinder: Doublet Detection in Single-Cell RNA Sequencing Data Using Artificial Nearest Neighbors. Cell Syst.

[38] Hirz T, Mei S, Sarkar H, Kfoury Y, Wu S, Verhoeven BM (2023). Dissecting the immune suppressive human prostate tumor microenvironment via integrated single-cell and spatial transcriptomic analyses. Nat Commun.

[39] Zhao Z, Ding Y, Tran LJ, Chai G, Lin L (2023). Innovative breakthroughs facilitated by single-cell multi-omics: manipulating natural killer cell functionality correlates with a novel subcategory of melanoma cells. Front Immunol.

[40] Zeng Z, Ma Y, Hu L, Tan B, Liu P, Wang Y (2024). OmicVerse: a framework for bridging and deepening insights across bulk and single-cell sequencing. Nat Commun.

[41] Chu T, Wang Z, Pe'er D, Danko CG (2022). Cell type and gene expression deconvolution with BayesPrism enables Bayesian integrative analysis across bulk and single-cell RNA sequencing in oncology. Nat Cancer.

[42] Li J, Pan X, Yuan Y, Shen H-B TFvelo: gene regulation inspired RNA velocity estimation. bioRxiv. 2023:2023.07.12.548785.

[43] Wolf FA, Hamey FK, Plass M, Solana J, Dahlin JS, Göttgens B (2019). PAGA: graph abstraction reconciles clustering with trajectory inference through a topology preserving map of single cells. Genome Biol.

[44] Patel AP, Tirosh I, Trombetta JJ, Shalek AK, Gillespie SM, Wakimoto H (2014). Single-cell RNA-seq highlights intratumoral heterogeneity in primary glioblastoma. Science.

[45] Aibar S, González-Blas CB, Moerman T, Huynh-Thu VA, Imrichova H, Hulselmans G (2017). SCENIC: single-cell regulatory network inference and clustering. Nat Methods.

[46] Jin S, Plikus MV, Nie Q (2025). CellChat for systematic analysis of cell-cell communication from single-cell transcriptomics. Nat Protoc.

[47] Cable DM, Murray E, Zou LS, Goeva A, Macosko EZ, Chen F (2022). Robust decomposition of cell type mixtures in spatial transcriptomics. Nat Biotechnol.

[48] Pham D, Tan X, Balderson B, Xu J, Grice LF, Yoon S (2023). Robust mapping of spatiotemporal trajectories and cell-cell interactions in healthy and diseased tissues. Nat Commun.

[49] Tanevski J, Flores ROR, Gabor A, Schapiro D, Saez-Rodriguez J (2022). Explainable multiview framework for dissecting spatial relationships from highly multiplexed data. Genome Biol.

[50] Yin Y, Xu L, Chang Y, Zeng T, Chen X, Wang A (2019). N-Myc promotes therapeutic resistance development of neuroendocrine prostate cancer by differentially regulating miR-421/ATM pathway. Mol Cancer.

[51] Ma W, Ge Q, Guan Y, Zhang L, Huang L, Chen L (2024). Integrated analysis of histone modification features in clear cell renal cancer for risk stratification and therapeutic prediction. Transl Oncol.

[52] O'Neill LA, Kishton RJ, Rathmell J (2016). A guide to immunometabolism for immunologists. Nat Rev Immunol.

[53] Mills EL, Kelly B, O'Neill LAJ (2017). Mitochondria are the powerhouses of immunity. Nat Immunol.

[54] Di Conza G, Tsai CH, Gallart-Ayala H, Yu YR, Franco F, Zaffalon L (2021). Tumor-induced reshuffling of lipid composition on the endoplasmic reticulum membrane sustains macrophage survival and pro-tumorigenic activity. Nat Immunol.

[55] Noy R, Pollard JW (2014). Tumor-associated macrophages: from mechanisms to therapy. Immunity.

[56] Deffner M, Schneider-Hohendorf T, Schulte-Mecklenbeck A, Falk S, Lu IN, Ostkamp P (2024). Chemokine-mediated cell migration into the central nervous system in progressive multifocal leukoencephalopathy. Cell Rep Med.

[57] Young MD, Mitchell TJ, Vieira Braga FA, Tran MGB, Stewart BJ, Ferdinand JR (2018). Single-cell transcriptomes from human kidneys reveal the cellular identity of renal tumors. Science.

[58] Zheng L, Qin S, Si W, Wang A, Xing B, Gao R (2021). Pan-cancer single-cell landscape of tumor-infiltrating T cells. Science.

[59] Wherry EJ, Kurachi M (2015). Molecular and cellular insights into T cell exhaustion. Nat Rev Immunol.

[60] Wang C, Li Y, Wang L, Han Y, Gao X, Li T (2024). SPP1 represents a therapeutic target that promotes the progression of oesophageal squamous cell carcinoma by driving M2 macrophage infiltration. Br J Cancer.

[61] Singh S, Gouri V, Samant M (2023). TGF-β in correlation with tumor progression, immunosuppression and targeted therapy in colorectal cancer. Med Oncol.

[62] Powles T, Albiges L, Bex A, Comperat E, Grünwald V, Kanesvaran R (2024). Renal cell carcinoma: ESMO Clinical Practice Guideline for diagnosis, treatment and follow-up. Ann Oncol.

[63] Gong J, Maia MC, Dizman N, Govindarajan A, Pal SK (2016). Metastasis in renal cell carcinoma: Biology and implications for therapy. Asian J Urol.

[64] Bi K, He MX, Bakouny Z, Kanodia A, Napolitano S, Wu J (2021). Tumor and immune reprogramming during immunotherapy in advanced renal cell carcinoma. Cancer Cell.

[65] Ghoreifi A, Vaishampayan U, Yin M, Psutka SP, Djaladat H (2024). Immune Checkpoint Inhibitor Therapy Before Nephrectomy for Locally Advanced and Metastatic Renal Cell Carcinoma: A Review. JAMA Oncol.

[66] Obradovic A, Chowdhury N, Haake SM, Ager C, Wang V, Vlahos L (2021). Single-cell protein activity analysis identifies recurrence-associated renal tumor macrophages. Cell.

[67] Phu TA, Ng M, Vu NK, Gao AS, Raffai RL (2023). ApoE expression in macrophages communicates immunometabolic signaling that controls hyperlipidemia-driven hematopoiesis & inflammation via extracellular vesicles. J Extracell Vesicles.

[68] Zhao Y, Yu Y, Li X, Guo A (2024). CCAAT enhancer binding protein delta activates vesicle associated membrane protein 3 transcription to enhance chemoresistance and extracellular PD-L1 expression in triple-negative breast cancer. J Exp Clin Cancer Res.

[69] Wei J, Marisetty A, Schrand B, Gabrusiewicz K, Hashimoto Y, Ott M (2019). Osteopontin mediates glioblastoma-associated macrophage infiltration and is a potential therapeutic target. J Clin Invest.

[70] Xu X, Lin J, Wang J, Wang Y, Zhu Y, Wang J (2024). SPP1 expression indicates outcome of immunotherapy plus tyrosine kinase inhibition in advanced renal cell carcinoma. Hum Vaccin Immunother.

[71] Batlle E, Massagué J (2019). Transforming Growth Factor-β Signaling in Immunity and Cancer. Immunity.

[72] Dongre A, Rashidian M, Eaton EN, Reinhardt F, Thiru P, Zagorulya M (2021). Direct and Indirect Regulators of Epithelial-Mesenchymal Transition-Mediated Immunosuppression in Breast Carcinomas. Cancer Discov.

[73] Hsiao YW, Li CF, Chi JY, Tseng JT, Chang Y, Hsu LJ (2013). CCAAT/enhancer binding protein δ in macrophages contributes to immunosuppression and inhibits phagocytosis in nasopharyngeal carcinoma. Sci Signal.

[74] Ko CY, Chang WC, Wang JM (2015). Biological roles of CCAAT/Enhancer-binding protein delta during inflammation. J Biomed Sci.

[75] Li Q, Zhao W, Wang W, Chen P, Niu Z, Ni S (2024). Anti-PD-1/TGF-βRII bispecific antibody fusion protein LBL-015 in patients with advanced malignant tumors: A phase I, first-in-human, open-label, multicenter, dose-escalation study. Journal of Clinical Oncology.

