# Supplementary material for: Spatially segregated APOE+ macrophages restrict immunotherapy efficacy in clear cell renal cell carcinoma
Source: Theranostics. 2025 Apr 13;15(11):5312–36. doi: 10.7150/thno.109097 (PMC12036886; doi:10.7150/thno.109097)
Supplement: Supplementary file 1 — Supplementary figures and tables. [file thnov15p5312s1.pdf]

Figure S1. Flowchart of this study.

Figure S2. Single-cell clustering, differential expression, and APOE survival analysis.

(**A**) UMAP visualization of 13 samples after batch effect correction. (**B**) UMAP showing 13 distinct cell clusters. (**C**) Differentially expressed genes (DEGs) across the 13 clusters. (**D**) Tissue preference of the ten cell types across samples. (**E**) Distribution of the ten cell types across individual samples. (**F**) Similarity between the ten cell types, calculated using Pearson's correlation coefficient. (**G**) Heatmap showing differential expression of the top ten DEGs in the ICB-resistant group from the CheckMate cohort. The bar density plot illustrates differential expression of APOE among ICB, clinical benefit (CB), and no clinical benefit (NCB) groups in the CheckMate cohort. (**H**) Kaplan-Meier plots demonstrating distinct overall survival (OS) and progression-free survival (PFS) outcomes for patients stratified by APOE expression, with the median value of APOE as the cutoff. (**I**) Comparison of ICB response rates between high and low APOE expression groups. CR: complete response; PR: partial response; PD: progressive disease; SD: stable disease.

Figure S3. GSVA enrichment and macrophage deconvolution in EMTAB3267 and TCGA-KIRC cohorts.

(**A**) Bar plot showing the Gene Set Enrichment Analysis (GSEA) results based on the Gene Ontology (GO) database (top) and the KEGG database (bottom). (**B**) Following the TIDE procedure, patients were classified into potential clinical benefit (CB) and no clinical benefit (NCB) groups, and their cellular components were inferred using the BayesPrim deconvolution algorithm. Heatmap displaying the differential distribution of six macrophage subsets between CB and NCB groups in the EMTAB3267 cohort. (**C**) Comparison of $APOE^{+}$ macrophage scores between CB and ICB groups in the EMTAB3267 cohort. (**D**) Based on the median APOE score, all patients were categorized into high-APOE+ and low-$APOE^{+}$ macrophage groups. A Kappa (κ) consistency test was conducted to assess the agreement between APOE+ macrophage groups and TIDE classifications. A Kappa value > 0.4 indicated favorable consistency. (**E**) Heatmap showing the differential distribution of six macrophage subsets between CB and NCB groups in the TCGA-KIRC cohort. (**F**) Comparison of $APOE^{+}$ macrophage scores between CB and ICB groups in the TCGA-KIRC cohort. (**G**) Kappa consistency test results for the TCGA-KIRC cohort.

Figure S4. SCENIC analysis of macrophage regulons.

(**A**) Top five regulons for each macrophage subset. (**B**) Differential regulon activity patterns across six macrophage subsets among the ICB groups. (**C**) Regulons of KLF2, KLF4, and KLF6, along with their associated target genes. (**D**) Regulons of the AP-1 complex (JUB, JUNB, FOS) and their target genes. (**E**) Regulons of AR, THAP1, SPI1, MLX, and DRAP1, along with their target genes. (**F**) Gene Ontology (GO) and KEGG enrichment analysis for target genes of the CEBPA, CEBPB, and CEBPD regulons. (**G**) Comparison of chemokine transcriptional activity (CXCL1, CXCL2, CCL4, CCL5, CCL7, CCL13, and CCL18) between the ICB groups. (H) Comparison of CCL4 and CCL5 expression levels between the ICB groups. * Represent $P < 0.05$; ** represent $P < 0.05$; *** represent $P < 0.05$. (**I**) Differential expression of CEBPD between ICB-sensitive and ICB-resistant patients based on the GSE67501 dataset (right) and the Miao et al. cohort (left). RE: responder; NR: non-responder; CB: clinical benefit; NCB: no clinical benefit.

Figure S5. Stlearn-based cell-cell communication analysis.

Top 50 ligand-receptor interactions among cell types based on Robust Cell Type Decomposition (RCTD) results.

Figure S6. The co-culture of CM and APOE neutralization on the effects of macrophage polarization and tumor progression in 769-P cells.

(**A**) A colony formation assay was performed to assess the impact of co-culture CM and APOE neutralization on the cloning ability of 769-P cells. The quantification and comparison of colony counts are depicted (* represent $P < 0.05$; ** represent $P < 0.01$; *** represent $P < 0.001$). (**B**) Transwell migration assays analysis showed the different migration ability of 769-P cells. The quantity of cells that migrated was measured. (**C**) Evaluation of the effects of co-culturing CM and APOE neutralization on cell migration by wound healing analysis. A quantitative assessment of the percentage of wound closure is presented. (**D**) Assess the impact of co-culturing CM and APOE neutralization on tumor cell proliferation by CCK-8 proliferation analysis.

Table S1. Detailed descriptions of the R and Python packages utilized, the study cohorts, and the antibodies employed are provided.

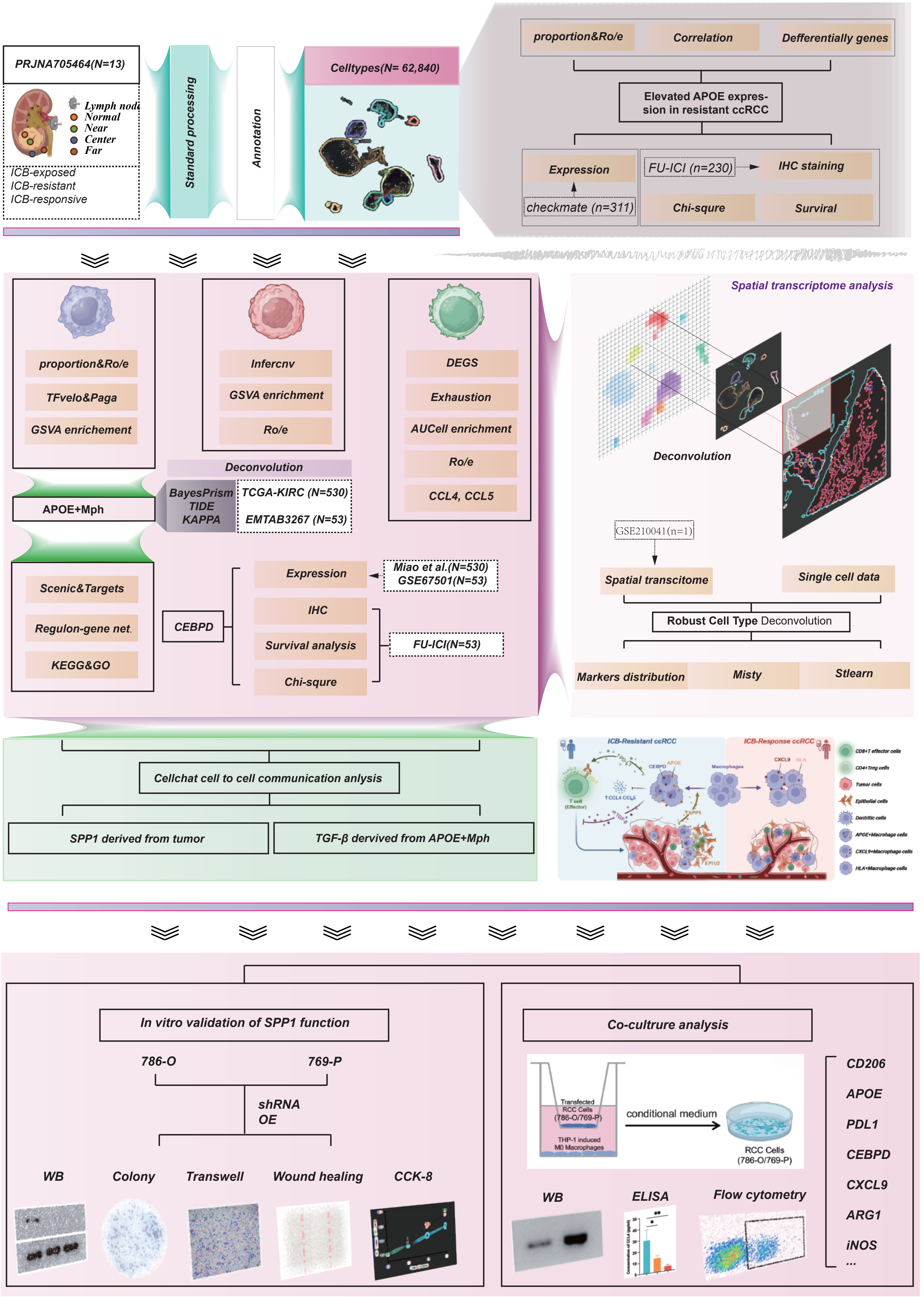

PRJNA705464(N=13)
Lymph node
Normal
Near
Center
Far
ICB-exposed
ICB-resistant
ICB-responsive
Standard processing
Annotation
Celltypes(N= 62,840)
proportion&Ro/e
Correlation
Defferentially genes
Elevated APOE expression in resistant ccRCC
Expression
checkmate (n=311)
FU-ICI (n=230)
IHC staining
Chi-squre
Surviral
proportion&Ro/e
TFvelo&Paga
GSVA enrichement
Infercnv
GSVA enrichment
Ro/e
DEGS
Exhaustion
AUCell enrichment
Ro/e
CCL4, CCL5
Deconvolution
BayesPrism
TIDE
KAPPA
TCGA-KIRC (N=530)
EMTAB3267 (N=53)
APOE+Mph
Scenic&Targets
Regulon-gene net.
KEGG&GO
CEBPD
Expression
Miao et al.(N=530)
GSE67501(N=53)
IHC
Survival analysis
FU-ICI(N=53)
Chi-squre
Spatial transcriptome analysis
Deconvolution
GSE210041(n=1)
Spatial transcitome
Single cell data
Robust Cell Type Deconvolution
Markers distribution
Misty
Stlearn
Cellchat cell to cell communication anlysis
SPP1 derived from tumor
TGF-β dervived from APOE+Mph
ICB-Resistant ccRCC
ICB-Response ccRCC
In vitro validation of SPP1 function
786-O
769-P
shRNA
OE
WB
Colony
Transwell
Wound healing
CCK-8
Co-cultrure analysis
Transfected RCC Cells (786-O/769-P)
THP-1 induced M0 Macrophages
conditional medium
RCC Cells (786-O/769-P)
WB
ELISA
Flow cytometry
CD206
APOE
PDL1
CEBPD
CXCL9
ARG1
iNOS
...

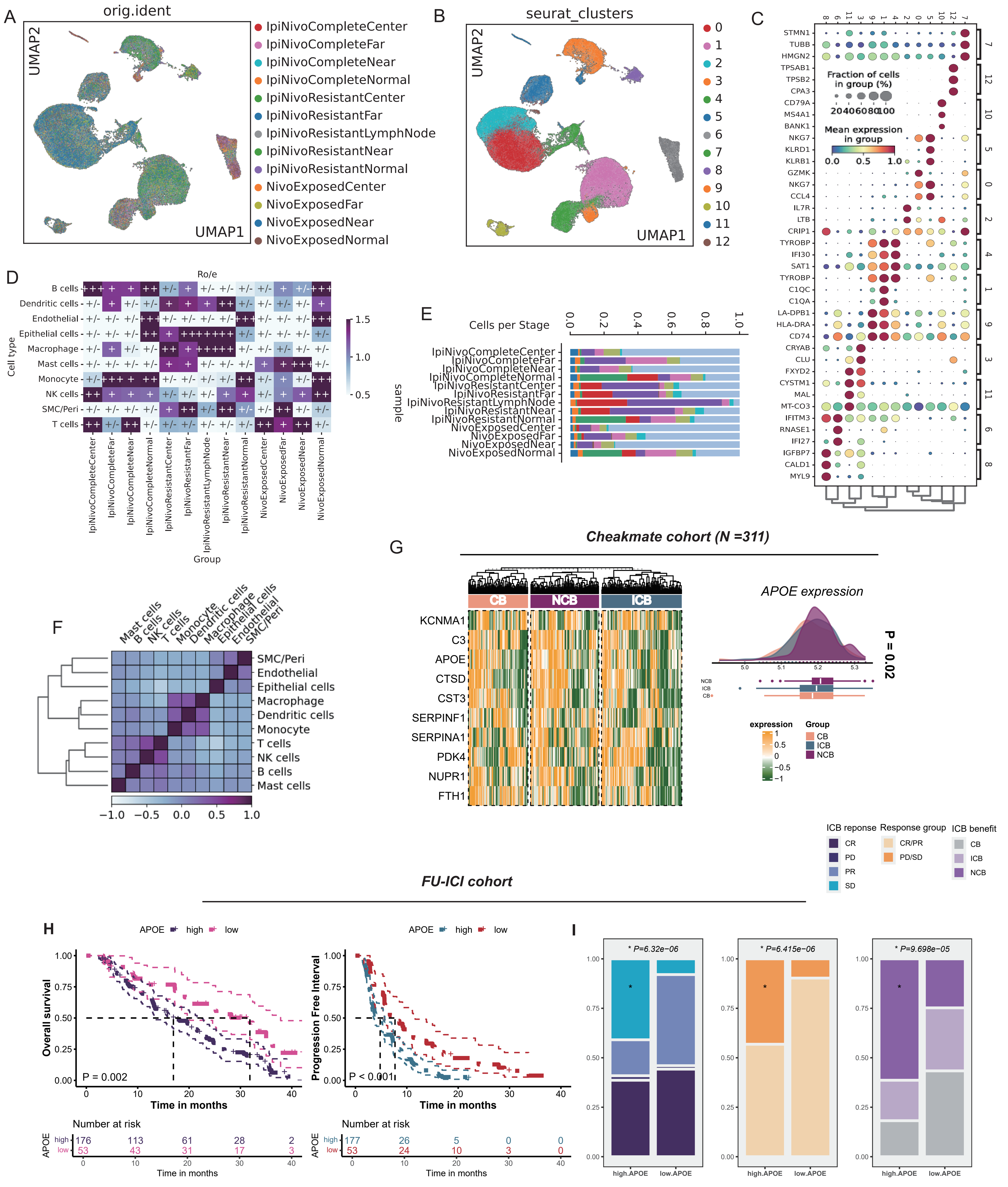

A
orig.ident
UMAP2
UMAP1
IpiNivoCompleteCenter
IpiNivoCompleteFar
IpiNivoCompleteNear
IpiNivoCompleteNormal
IpiNivoResistantCenter
IpiNivoResistantFar
IpiNivoResistantLymphNode
IpiNivoResistantNear
IpiNivoResistantNormal
NivoExposedCenter
NivoExposedFar
NivoExposedNear
NivoExposedNormal
B
seurat_clusters
0 1 2 3 4 5 6 7 8 9 10 11 12
C
Fraction of cells in group (%)
20 40 60 80 100
Mean expression in group
0.0 0.5 1.0
STMN1 TUBB HMGN2 TPSAB1 TPSB2 CPA3 CD79A MS4A1 BANK1 NKG7 KLRD1 KLRB1 GZMK NKG7 CCL4 IL7R LTB CRIP1 TYROBP IFI30 SAT1 TYROBP C1QC C1QA LA-DPB1 HLA-DRA CD74 CRYAB CLU FXYD2 CYSTM1 MAL MT-CO3 IFITM3 RNASE1 IFI27 IGFBP7 CALD1 MYL9
D
Ro/e
Cell type
B cells
Dendritic cells
Endothelial
Epithelial cells
Macrophage
Mast cells
Monocyte
NK cells
SMC/Peri
T cells
Group
1.5
1.0
0.5
E
Cells per Stage
sample
0.0 0.2 0.4 0.6 0.8 1.0
F
Mast cells
B cells
NK cells
T cells
Monocyte
Dendritic cells
Macrophage
Epithelial cells
Endothelial
SMC/Peri
−1.0 −0.5 0.0 0.5 1.0
G
Cheakmate cohort (N =311)
CB NCB ICB
KCNMA1
C3
APOE
CTSD
CST3
SERPINF1
SERPINA1
PDK4
NUPR1
FTH1
APOE expression
P = 0.02
5.0 5.1 5.2 5.3
NCB
ICB
CB
expression
1
0.5
0
−0.5
−1
Group
CB
ICB
NCB
ICB reponse
CR
PD
PR
SD
Response group
CR/PR
PD/SD
ICB benefit
CB
ICB
NCB
FU-ICI cohort
H
APOE high low
Overall survival
Time in months
P = 0.002
Number at risk
APOE
high 176 113 61 28 2
low 53 43 31 17 3
0 10 20 30 40
Progression Free Interval
P < 0.001
high 177 26 5 0 0
low 53 24 10 3 0
I
* P=6.32e−06
* P=6.415e−06
* P=9.698e−05
high.APOE
low.APOE

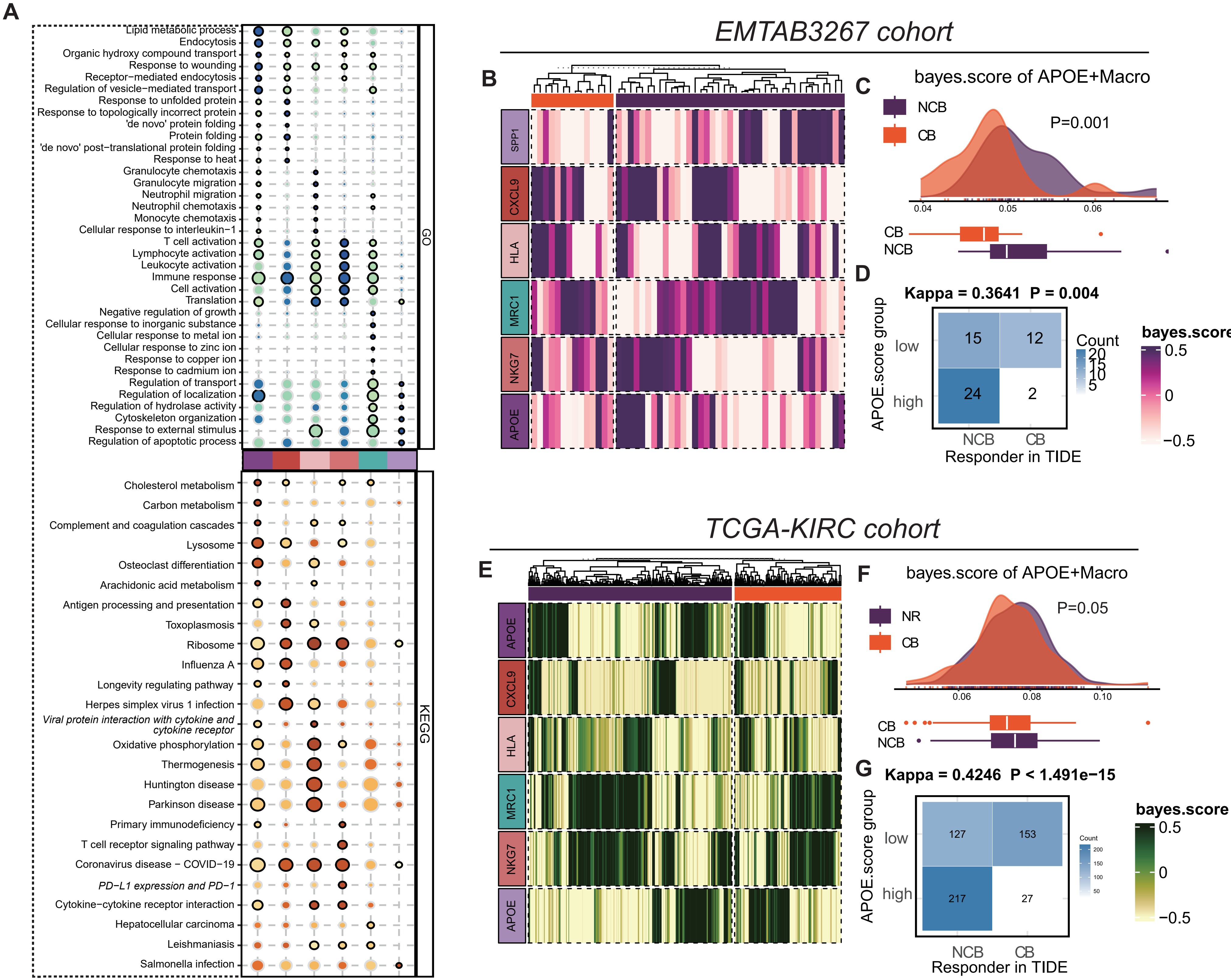
A
Lipid metabolic process
Endocytosis
Organic hydroxy compound transport
Response to wounding
Receptor−mediated endocytosis
Regulation of vesicle−mediated transport
Response to unfolded protein
Response to topologically incorrect protein
'de novo' protein folding
Protein folding
'de novo' post−translational protein folding
Response to heat
Granulocyte chemotaxis
Granulocyte migration
Neutrophil migration
Neutrophil chemotaxis
Monocyte chemotaxis
Cellular response to interleukin−1
T cell activation
Lymphocyte activation
Leukocyte activation
Immune response
Cell activation
Translation
Negative regulation of growth
Cellular response to inorganic substance
Cellular response to metal ion
Cellular response to zinc ion
Response to copper ion
Response to cadmium ion
Regulation of transport
Regulation of localization
Regulation of hydrolase activity
Cytoskeleton organization
Response to external stimulus
Regulation of apoptotic process
GO
Cholesterol metabolism
Carbon metabolism
Complement and coagulation cascades
Lysosome
Osteoclast differentiation
Arachidonic acid metabolism
Antigen processing and presentation
Toxoplasmosis
Ribosome
Influenza A
Longevity regulating pathway
Herpes simplex virus 1 infection
Viral protein interaction with cytokine and cytokine receptor
Oxidative phosphorylation
Thermogenesis
Huntington disease
Parkinson disease
Primary immunodeficiency
T cell receptor signaling pathway
Coronavirus disease − COVID−19
PD−L1 expression and PD−1
Cytokine−cytokine receptor interaction
Hepatocellular carcinoma
Leishmaniasis
Salmonella infection
KEGG
EMTAB3267 cohort
B
SPP1
CXCL9
HLA
MRC1
NKG7
APOE
C
bayes.score of APOE+Macro
NCB
CB
P=0.001
0.04
0.05
0.06
CB
NCB
D
Kappa = 0.3641 P = 0.004
APOE.score group
low
high
15
12
24
2
NCB
CB
Responder in TIDE
Count
20
15
10
5
bayes.score
0.5
0
−0.5
TCGA-KIRC cohort
E
APOE
CXCL9
HLA
MRC1
NKG7
APOE
F
bayes.score of APOE+Macro
NR
CB
P=0.05
0.06
0.08
0.10
CB
NCB
G
Kappa = 0.4246 P < 1.491e−15
APOE.score group
low
high
127
153
217
27
Count
200
150
100
50
NCB
CB
Responder in TIDE
bayes.score
0.5
0
−0.5

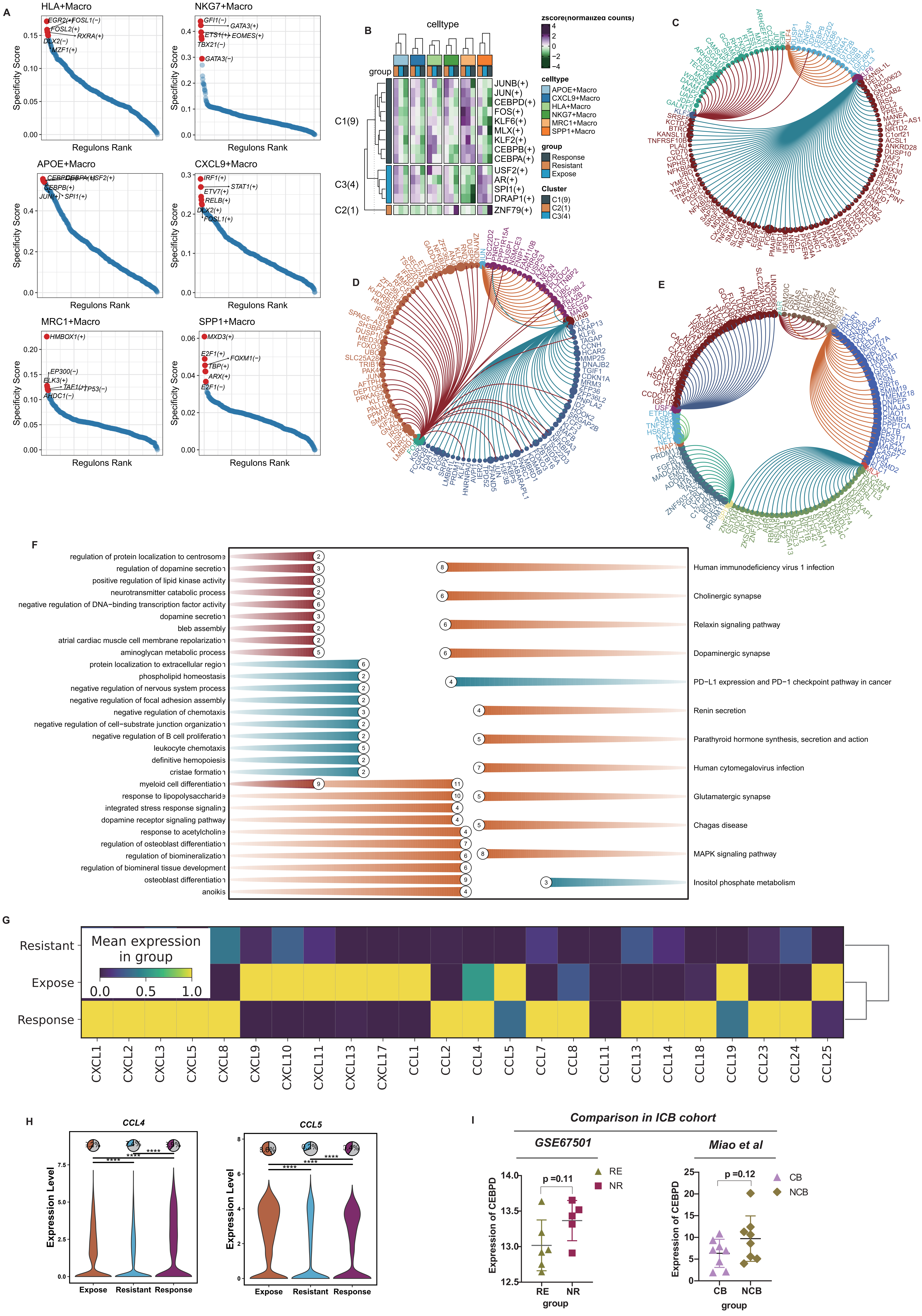
A
HLA+Macro
EGR2(+) FOSL1(−)
FOSL2(+)
RXRA(+)
DLX2(−)
MZF1(+)
NKG7+Macro
GFI1(−)
GATA3(+)
ETS1(+) EOMES(+)
TBX21(−)
GATA3(−)
APOE+Macro
CEBPB(+)
JUN(+) SPI1(+)
CXCL9+Macro
IRF1(+)
STAT1(+)
ETV7(+)
RELB(+)
DLX2(+)
FOSL1(+)
MRC1+Macro
HMBOX1(+)
EP300(−)
ELK3(+)
TAF1(+) TP53(−)
AHDC1(−)
SPP1+Macro
MXD3(+)
E2F1(+)
FOXM1(−)
TBP(+)
ARX(+)
E2F1(−)
Specificity Score
Regulons Rank
B
celltype
group
zscore(normalized counts)
C1(9)
C3(4)
C2(1)
JUNB(+)
JUN(+)
CEBPD(+)
FOS(+)
KLF6(+)
MLX(+)
KLF2(+)
CEBPB(+)
CEBPA(+)
USF2(+)
AR(+)
SPI1(+)
DRAP1(+)
ZNF79(+)
celltype
APOE+Macro
CXCL9+Macro
HLA+Macro
NKG7+Macro
MRC1+Macro
SPP1+Macro
group
Response
Resistant
Expose
Cluster
C1(9)
C2(1)
C3(4)
C
D
E
F
regulation of protein localization to centrosome
regulation of dopamine secretion
positive regulation of lipid kinase activity
neurotransmitter catabolic process
negative regulation of DNA−binding transcription factor activity
dopamine secretion
bleb assembly
atrial cardiac muscle cell membrane repolarization
aminoglycan metabolic process
protein localization to extracellular region
phospholipid homeostasis
negative regulation of nervous system process
negative regulation of focal adhesion assembly
negative regulation of chemotaxis
negative regulation of cell−substrate junction organization
negative regulation of B cell proliferation
leukocyte chemotaxis
definitive hemopoiesis
cristae formation
myeloid cell differentiation
response to lipopolysaccharide
integrated stress response signaling
dopamine receptor signaling pathway
response to acetylcholine
regulation of osteoblast differentiation
regulation of biomineralization
regulation of biomineral tissue development
osteoblast differentiation
anoikis
Human immunodeficiency virus 1 infection
Cholinergic synapse
Relaxin signaling pathway
Dopaminergic synapse
PD−L1 expression and PD−1 checkpoint pathway in cancer
Renin secretion
Parathyroid hormone synthesis, secretion and action
Human cytomegalovirus infection
Glutamatergic synapse
Chagas disease
MAPK signaling pathway
Inositol phosphate metabolism
G
Mean expression in group
0.0 0.5 1.0
Resistant
Expose
Response
CXCL1 CXCL2 CXCL3 CXCL5 CXCL8 CXCL9 CXCL10 CXCL11 CXCL13 CXCL17 CCL1 CCL2 CCL4 CCL5 CCL7 CCL8 CCL11 CCL13 CCL14 CCL18 CCL19 CCL23 CCL24 CCL25
H
CCL4
CCL5
Expression Level
Expose
Resistant
Response
I
Comparison in ICB cohort
GSE67501
Miao et al
p =0.11
p =0.12
RE
NR
CB
NCB
Expression of CEBPD
group

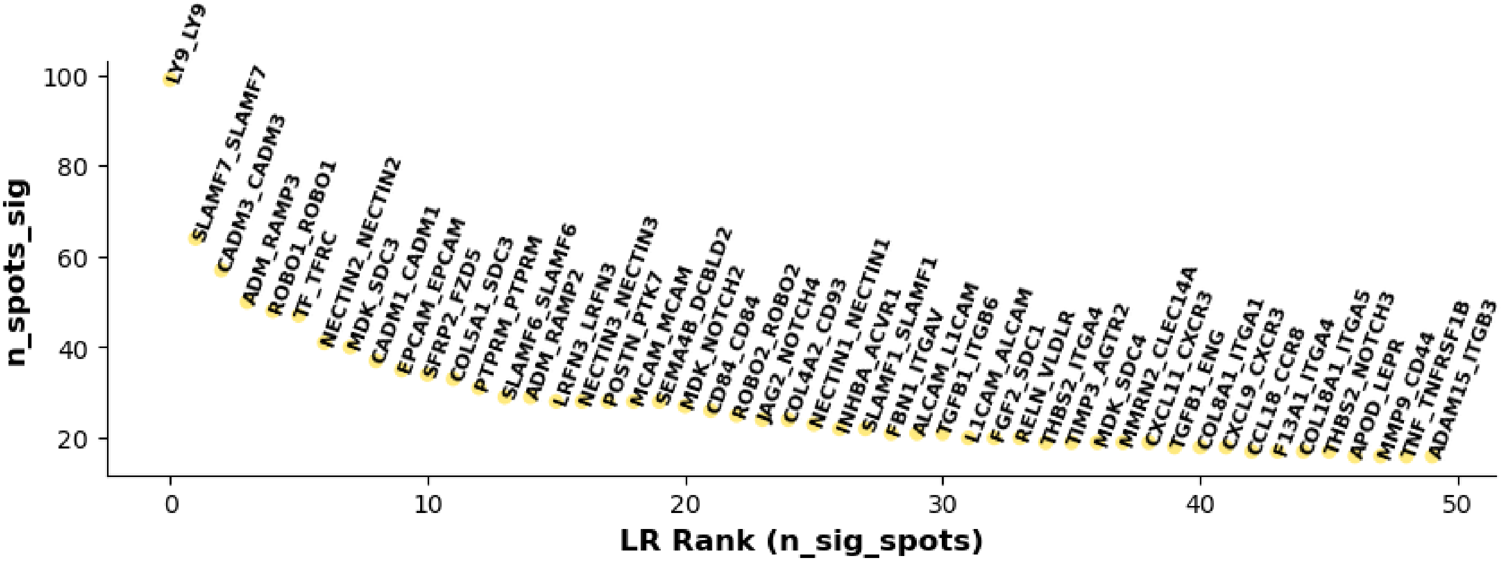
n_spots_sig
100
80
60
40
20
0
10
20
30
40
50
LR Rank (n_sig_spots)
LY9_LY9
SLAMF7_SLAMF7
CADM3_CADM3
ADM_RAMP3
ROBO1_ROBO1
TF_TFRC
NECTIN2_NECTIN2
MDK_SDC3
CADM1_CADM1
EPCAM_EPCAM
SFRP2_FZD5
COL5A1_SDC3
PTPRM_PTPRM
SLAMF6_SLAMF6
ADM_RAMP2
LRFN3_LRFN3
NECTIN3_NECTIN3
POSTN_PTK7
MCAM_MCAM
SEMA4B_DCBLD2
MDK_NOTCH2
CD84_CD84
ROBO2_ROBO2
JAG2_NOTCH4
COL4A2_CD93
NECTIN1_NECTIN1
INHBA_ACVR1
SLAMF1_SLAMF1
FBN1_ITGAV
ALCAM_L1CAM
TGFB1_ITGB6
L1CAM_ALCAM
FGF2_SDC1
RELN_VLDLR
THBS2_ITGA4
TIMP3_AGTR2
MDK_SDC4
MMRN2_CLEC14A
CXCL11_CXCR3
TGFB1_ENG
COL8A1_ITGA1
CXCL9_CXCR3
CCL18_CCR8
F13A1_ITGA4
COL18A1_ITGA5
THBS2_NOTCH3
APOD_LEPR
MMP9_CD44
TNF_TNFRSF1B
ADAM15_ITGB3

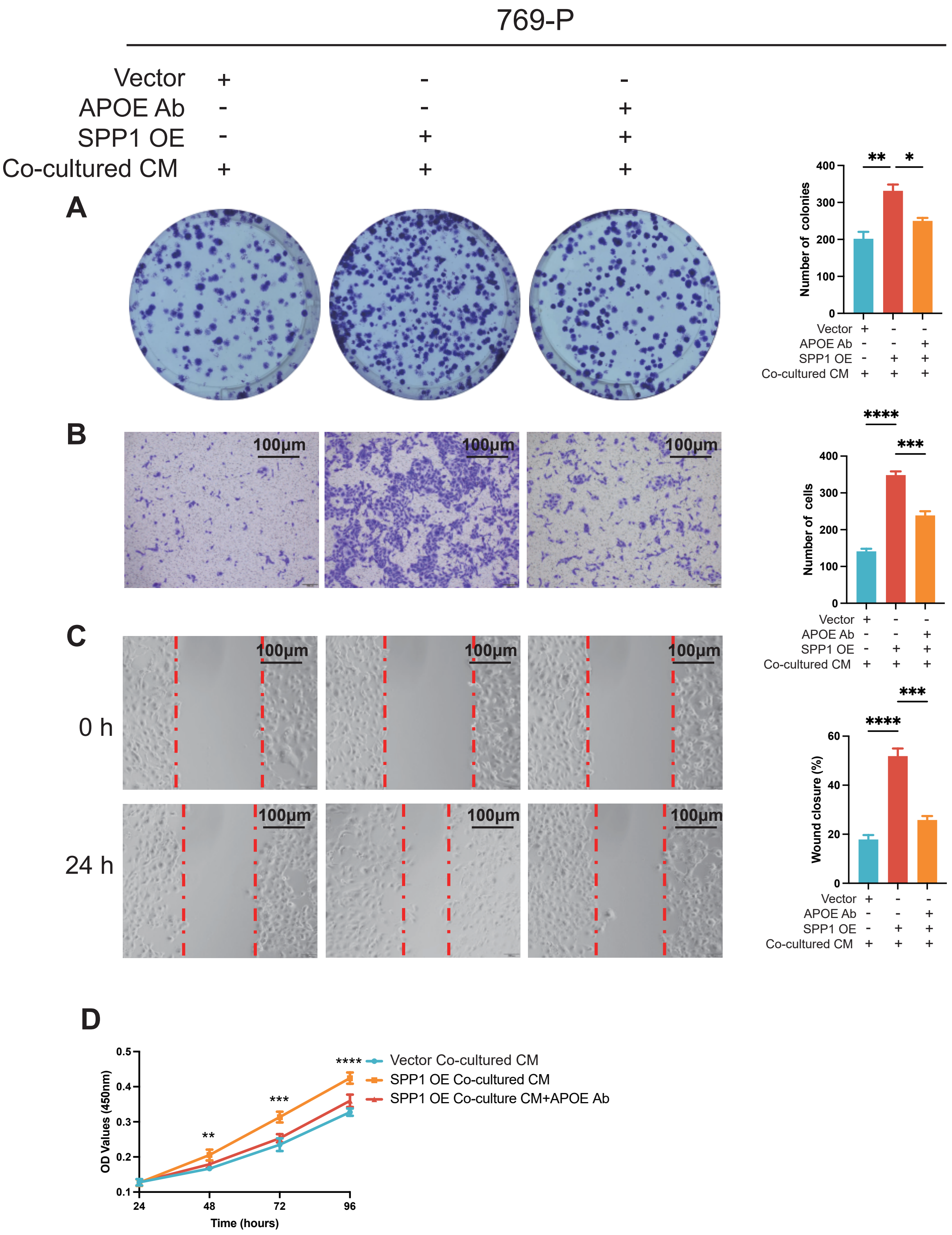

769-P
Vector + - -
APOE Ab - - +
SPP1 OE - + +
Co-cultured CM + + +
A
B
C
D
100μm
0 h
24 h
Number of colonies
Number of cells
Wound closure (%)
OD Values (450nm)
Time (hours)
Vector Co-cultured CM
SPP1 OE Co-cultured CM
SPP1 OE Co-culture CM+APOE Ab

Software and algorithms

Package	Version	
**R V4.2.2**	R Core Team	https://www.r-project.org/
**PythonV3.9**	Python Software Foundation	https://www.python.org/
**Seurat V4.3.0**	Stuart et al.(1)	https://satijalab.org/seurat/
**DoubletFinder**	McGinnis et al. (2)	https://github.com/chris-mcginnis-ucsf/DoubletFinder
**Harmony V0.1.0**	Korsunsky et al.(3)	https://cran.r-project.org/web/packages/harmony/index.html
**SCP**	Zhang et al.	https://github.com/zhanghao-njmu/SCP
**omicverse**	Zeng et al.(4)	https://omicverse.readthedocs.io/)
**Harmony V0.1.0**	Korsunsky et al.(3)	https://cran.r-project.org/web/packages/harmony/index.html
**clusterprofiler V4.0.5**	Yu et al.(5)	https://guangchuangyu.github.io/software/clusterProfiler/
**ggplot2 V3.3.5**	Wickham et al. (6)	https://ggplot2.tidyverse.org/
**AUCell V3.18**	Aibar et al.(7)	https://bioconductor.org/packages/release/bioc/html/AUCell.html
**BayesPrism v2.2**	Chu et al.(8)	https://github.com/Danko-Lab/BayesPrism/blob/main/tutorial_deconvolution.html
**TFvelco**	Li et al.(9)	https://github.com/xiaoyeye/TFvelo/tree/main/TFvelo
**PAGA**	Wolf et al.(10)	https://github.com/theislab/paga
**inferCNV (v1.6.0)**	Trinity CTAT Project	https://github.com/broadinstitute/inferCNV
**SCENIC (version 1.2.4)**	Aibar et al.(7)	https://scenic.aertslab.org/
**Cellchat (v.2.0)**	Jin et al.(11)	https://github.com/sqjin/CellChat/tree/master/tutorial
**MISTy (v1.2.1)**	Tanevski et al.(12)	https://github.com/saezlab/misty
**RCTD**	Cable et.al. (13)	https://github.com/dmcable/spacexr/blob/master/resources/RCTD_0.1.0.tar.gz
**Stlearn**	Duy et al.(14)	https://github.com/BiomedicalMachineLearning/stLearn
**TIDE**	Jing et al.	http://tide.dfci.harvard.edu/
**SCRNAtools**	Zhang et al.	https://github.com/scRNA-tools/scRNA-tools
**jjvocanlno**	Zhang et al.	https://github.com/junjunlab/scRNAtoolVis-manual/blob/main/jjvolcano.html
**Plot1cell**	Wu et al. (15)	https://github.com/TheHumphreysLab/plot1cell
**sva**	Leek et al. (16)	https://github.com/jtleek/sva-devel/blob/master/R/ComBat.R

**Ro/e**	Zh et al. (17)	https://rdrr.io/github/zhupinglab/Aodav/

Reference

1. Hao Y, Hao S, Andersen-Nissen E, Mauck WM, 3rd, Zheng S, Butler A, et al. Integrated analysis of multimodal single-cell data. Cell. 2021;184(13):3573-87.e29.
2. McGinnis CS, Murrow LM, Gartner ZJ. DoubletFinder: Doublet Detection in Single-Cell RNA Sequencing Data Using Artificial Nearest Neighbors. Cell Syst. 2019;8(4):329-37.e4.
3. Korsunsky I, Millard N, Fan J, Slowikowski K, Zhang F, Wei K, et al. Fast, sensitive and accurate integration of single-cell data with Harmony. Nat Methods. 2019;16(12):1289-96.
4. Zeng Z, Ma Y, Hu L, Tan B, Liu P, Wang Y, et al. OmicVerse: a framework for bridging and deepening insights across bulk and single-cell sequencing. Nat Commun. 2024;15(1):5983.
5. Yu G, Wang LG, Han Y, He QY. clusterProfiler: an R package for comparing biological themes among gene clusters. Omics. 2012;16(5):284-7.
6. Ginestet C. ggplot2: Elegant Graphics for Data Analysis. Journal of the Royal Statistical Society Series A: Statistics in Society. 2011;174(1):245-6.
7. Aibar S, González-Blas CB, Moerman T, Huynh-Thu VA, Imrichova H, Hulselmans G, et al. SCENIC: single-cell regulatory network inference and clustering. Nat Methods. 2017;14(11):1083-6.
8. Chu T, Wang Z, Pe'er D, Danko CG. Cell type and gene expression deconvolution with BayesPrism enables Bayesian integrative analysis across bulk and single-cell RNA sequencing in oncology. Nat Cancer. 2022;3(4):505-17.
9. Li J, Pan X, Yuan Y, Shen HB. TFvelo: gene regulation inspired RNA velocity estimation. Nat Commun. 2024;15(1):1387.
10. Wolf FA, Hamey FK, Plass M, Solana J, Dahlin JS, Göttgens B, et al. PAGA: graph abstraction reconciles clustering with trajectory inference through a topology preserving map of single cells. Genome Biol. 2019;20(1):59.
11. Jin S, Plikus MV, Nie Q. CellChat for systematic analysis of cell-cell communication from single-cell transcriptomics. Nat Protoc. 2025;20(1):180-219.
12. Tanevski J, Flores ROR, Gabor A, Schapiro D, Saez-Rodriguez J. Explainable multiview framework for dissecting spatial relationships from highly multiplexed data. Genome Biol. 2022;23(1):97.
13. Cable DM, Murray E, Zou LS, Goeva A, Macosko EZ, Chen F, et al. Robust decomposition of cell type mixtures in spatial transcriptomics. Nat Biotechnol. 2022;40(4):517-26.
14. Pham D, Tan X, Balderson B, Xu J, Grice LF, Yoon S, et al. Robust mapping of spatiotemporal trajectories and cell-cell interactions in healthy and diseased tissues. Nat Commun. 2023;14(1):7739.
15. Wu H, Gonzalez Villalobos R, Yao X, Reilly D, Chen T, Rankin M, et al. Mapping the single-cell transcriptomic response of murine diabetic kidney disease to therapies. Cell Metab. 2022;34(7):1064-78.e6.
16. Leek JT, Johnson WE, Parker HS, Jaffe AE, Storey JD. The sva package for removing batch effects and other unwanted variation in high-throughput experiments. Bioinformatics. 2012;28(6):882-3.
17. Zhang L, Yu X, Zheng L, Zhang Y, Li Y, Fang Q, et al. Lineage tracking reveals dynamic relationships of T cells in colorectal cancer. Nature. 2018;564(7735):268-72.

Data source

**TCGA-KIRC**	**530**	**https://portal.gdc.cancer.gov/**
**EMTAB3267**	53	https://www.ebi.ac.uk/arrayexpress/
**GSE67501**	11	https://www.ncbi.nlm.nih.gov/geo/
**Checkmate**	311	https://clinicaltrials.gov/
**Miao et al.**	16	https://pmc.ncbi.nlm.nih.gov/articles/PMC6035749/
**FU-ICI**	230	https://pubmed.ncbi.nlm.nih.gov/38040418/
**GSE210041**	2	https://www.ncbi.nlm.nih.gov/geo/
**PRJNA705464**	13	https://www.ebi.ac.uk/ena/browser/home

Antibody

Antibody	SOURCE	IDENTIFIER
**APOE**	Abcam	ab183597
**PD-L1**	Abcam	ab205921
**CEBPD**	Abcam	ab245214
**SPP1**	Abcam	ab218237
**ARG1**	Abcam	ab315110
**GAPDH**	Abcam	ab9485
**INOS**	Abcam	ab178945
**CXCL9**	Abcam	ab263442
**CD206**	Abcam	ab270647
